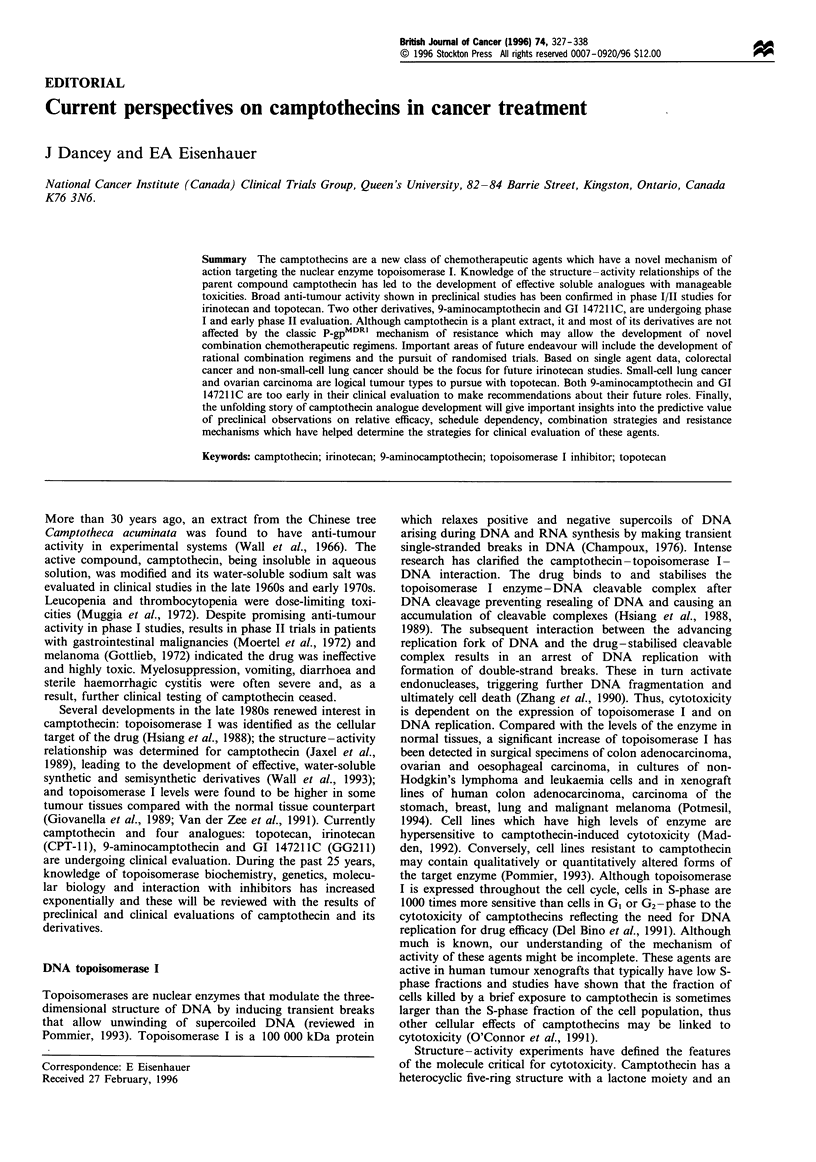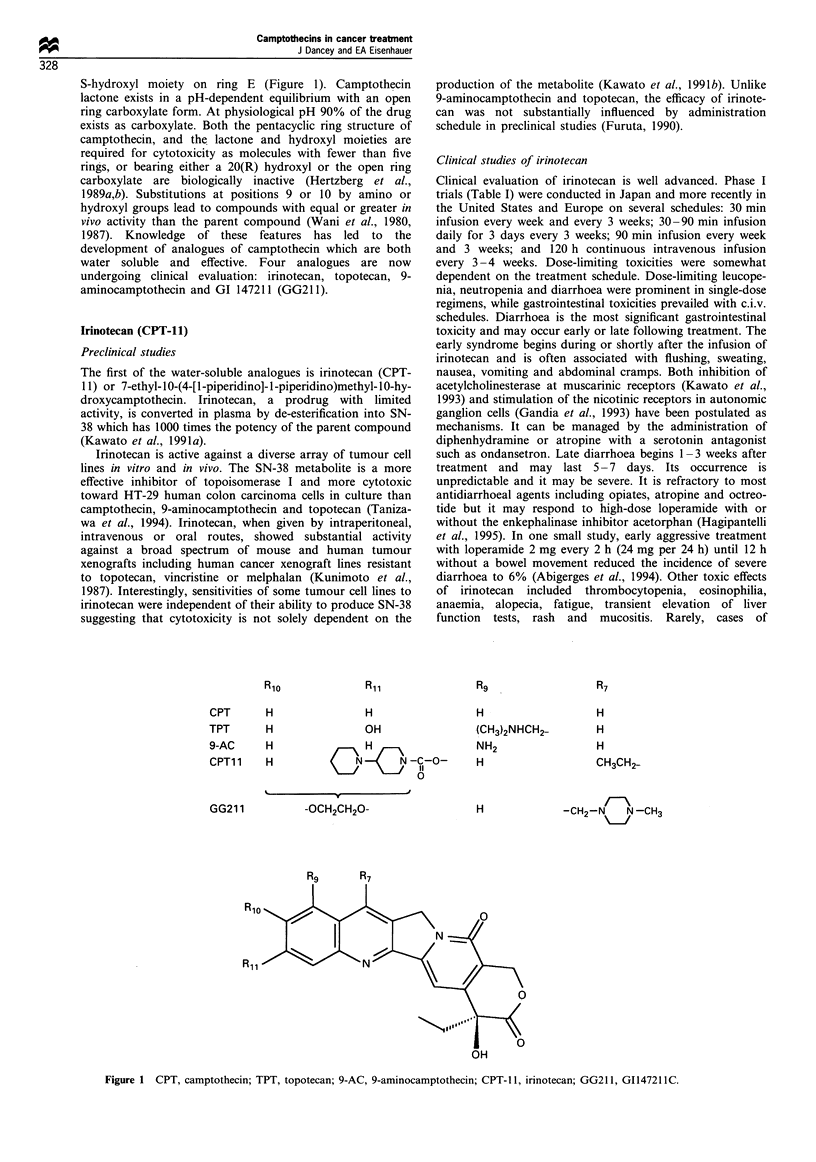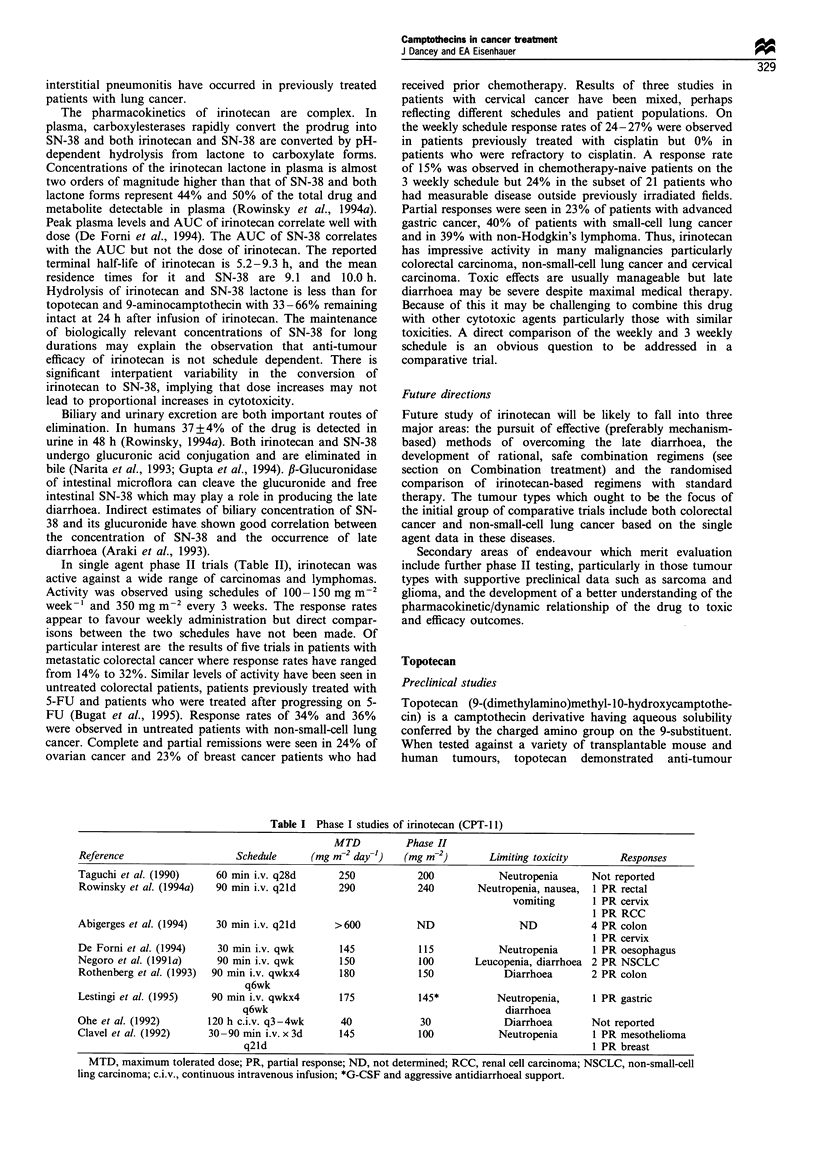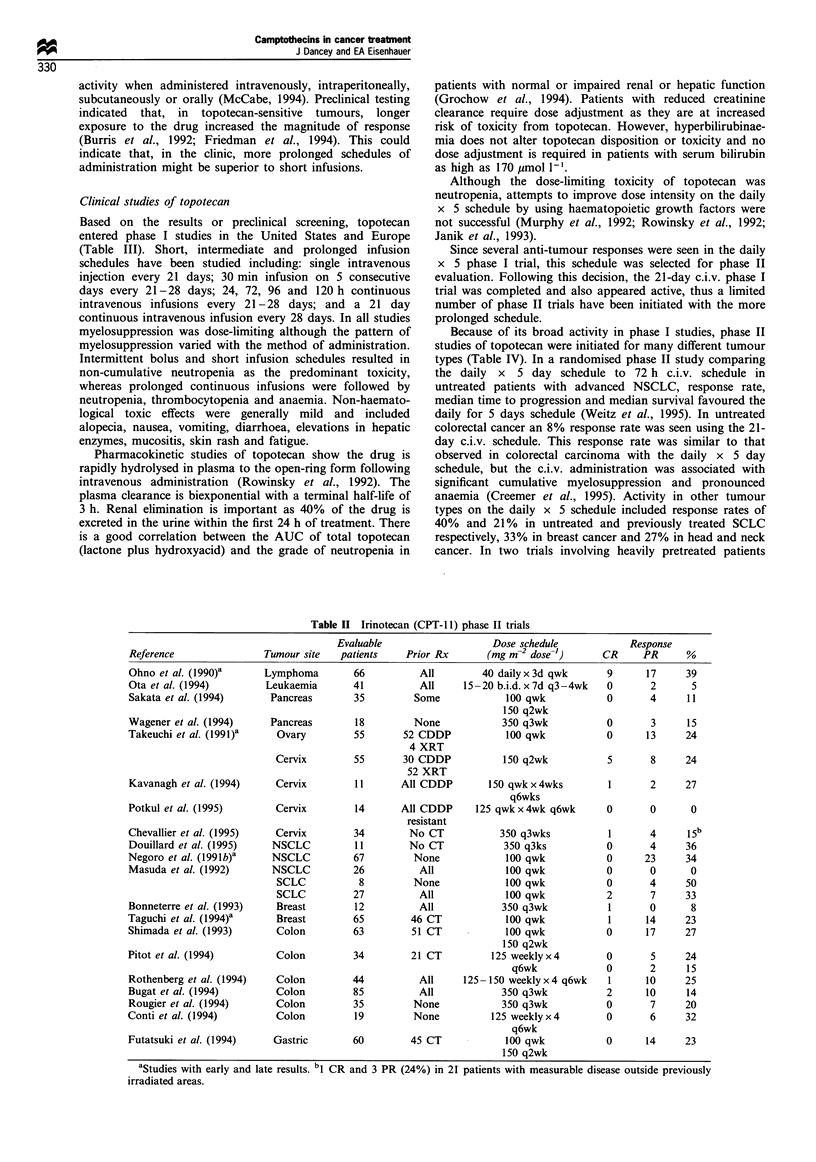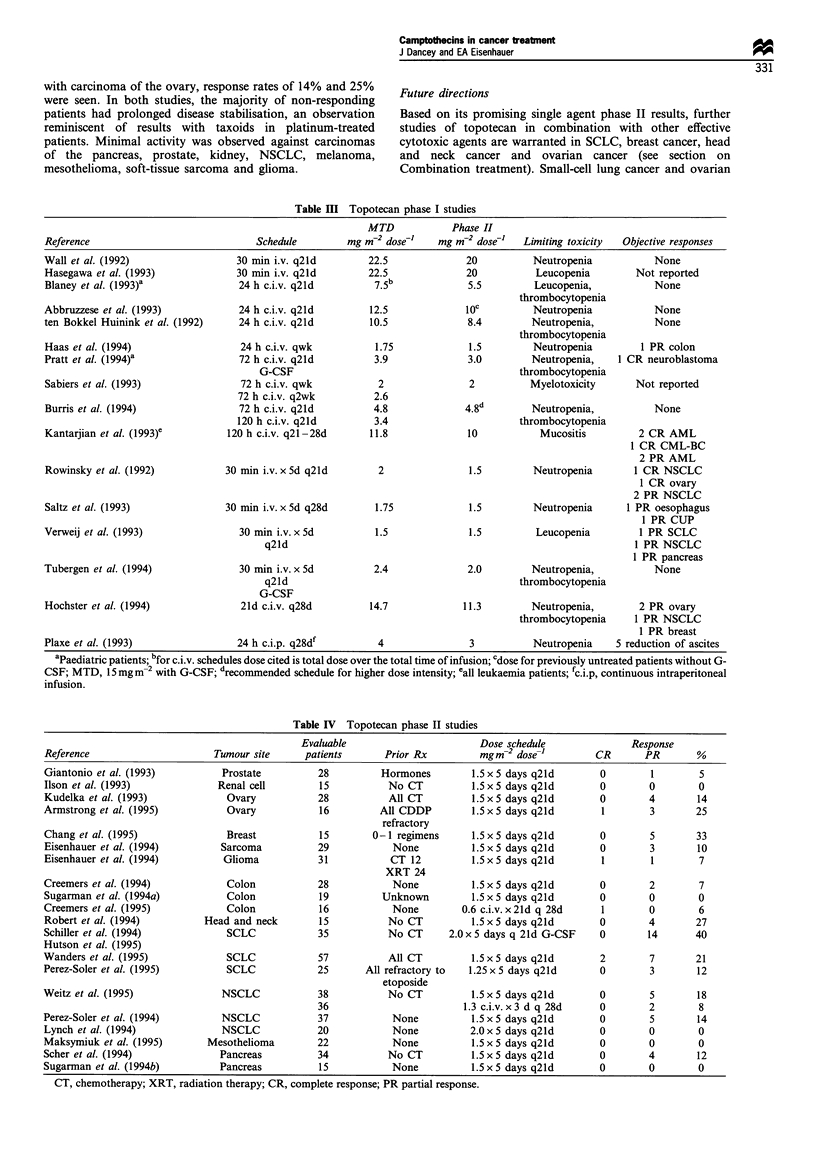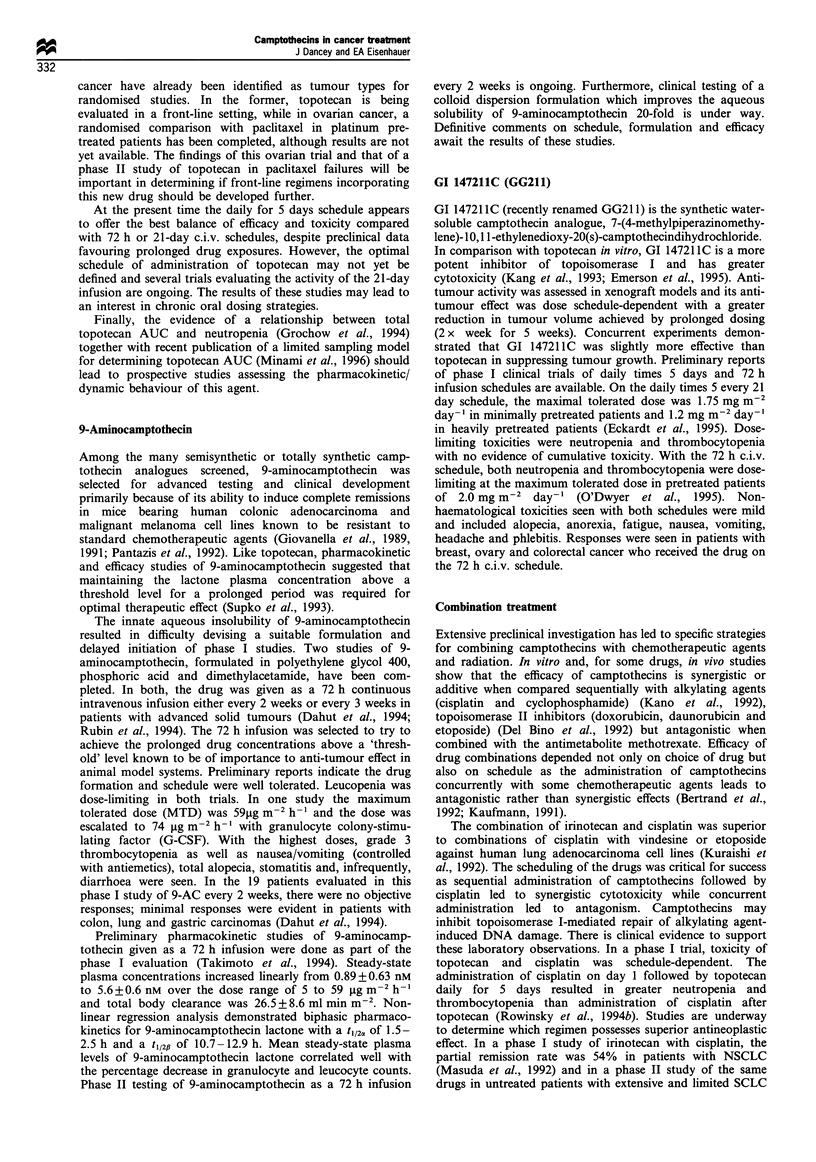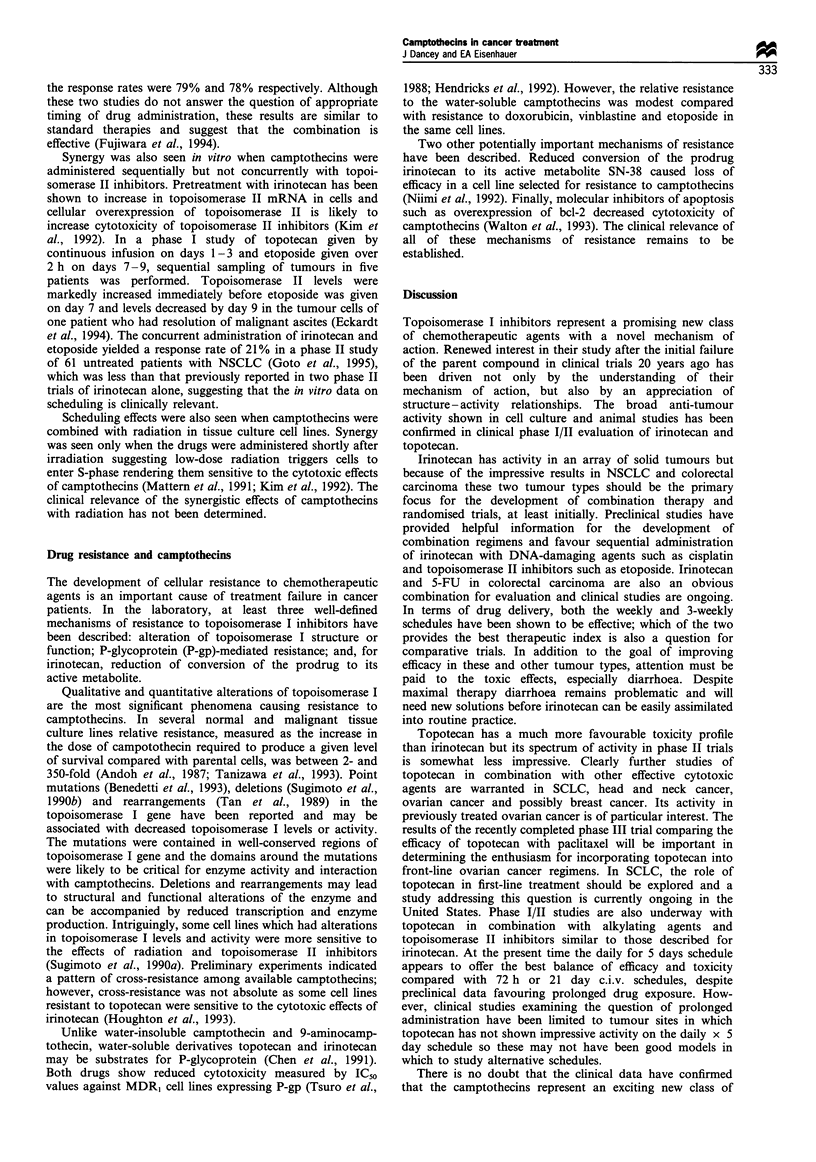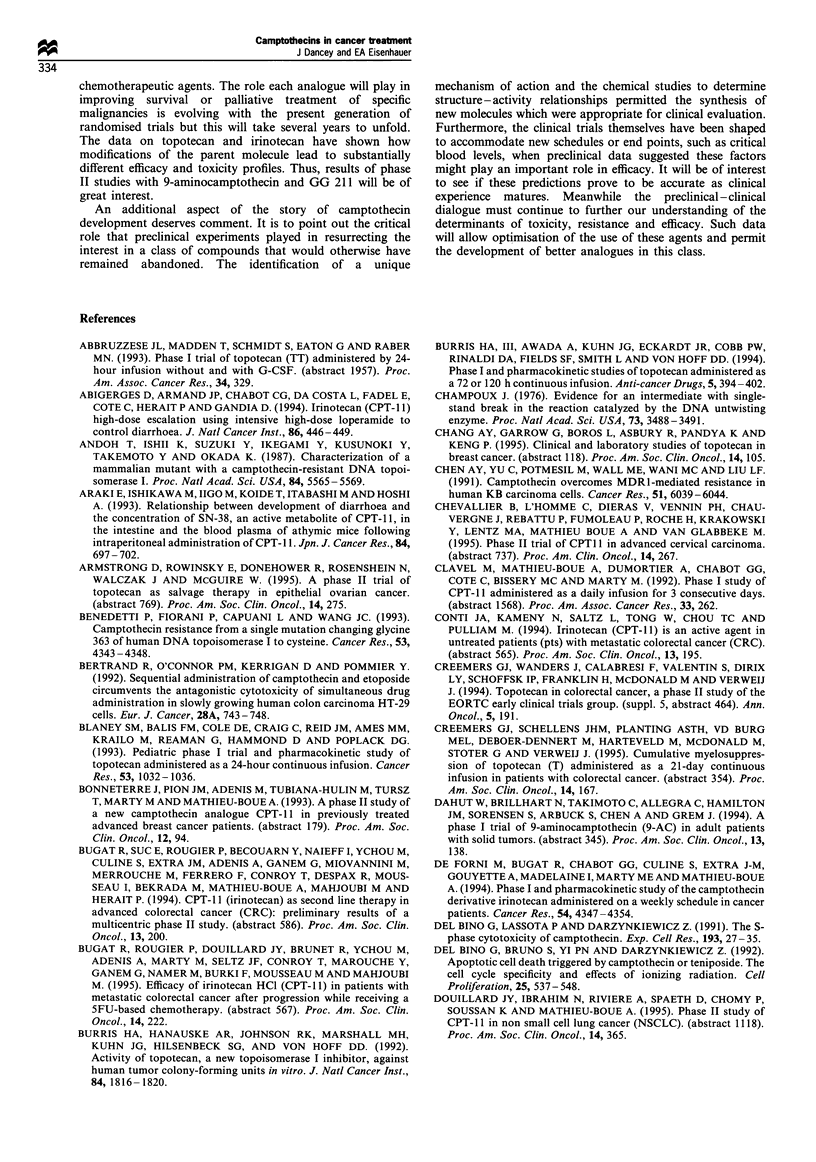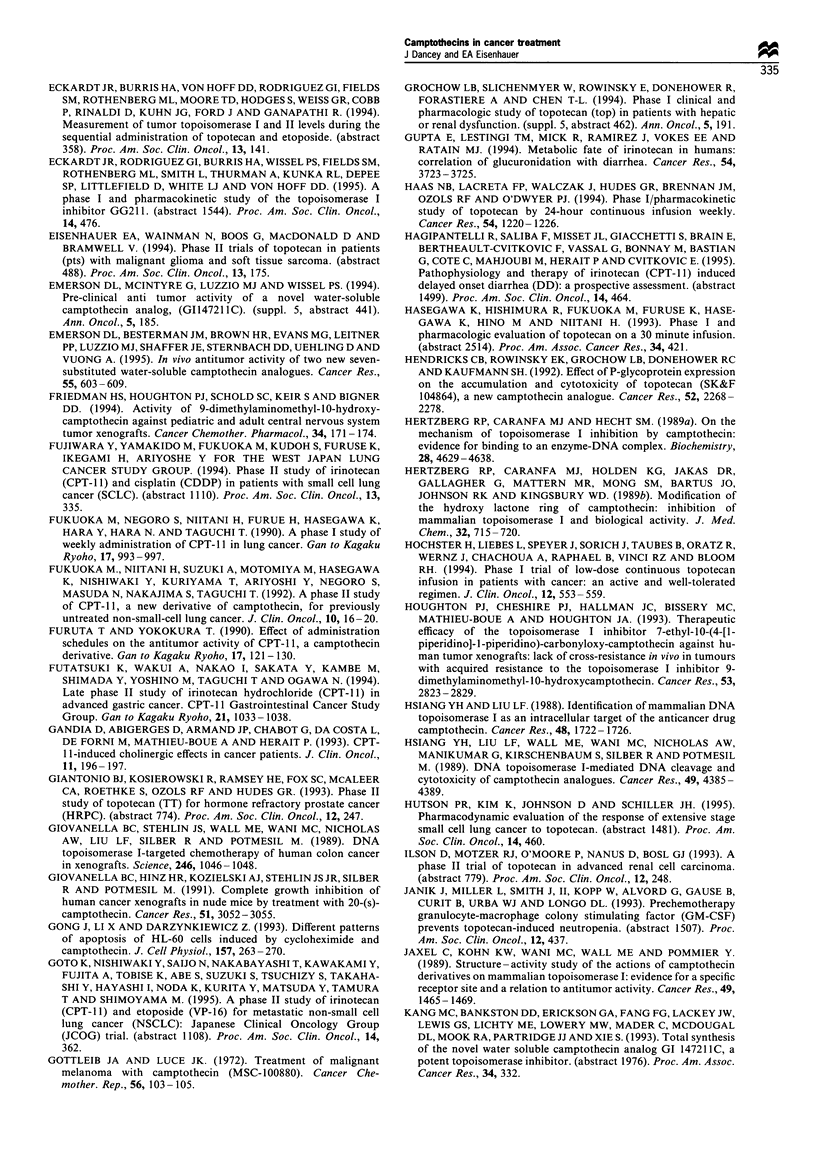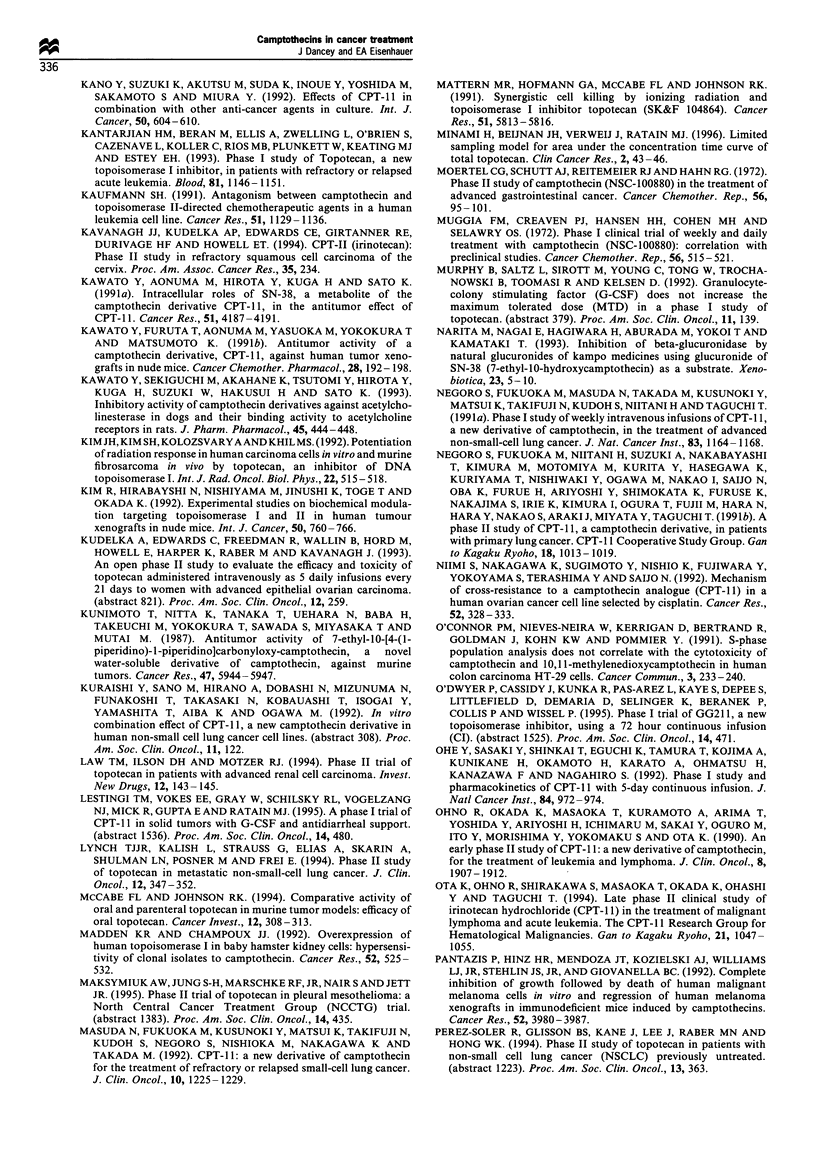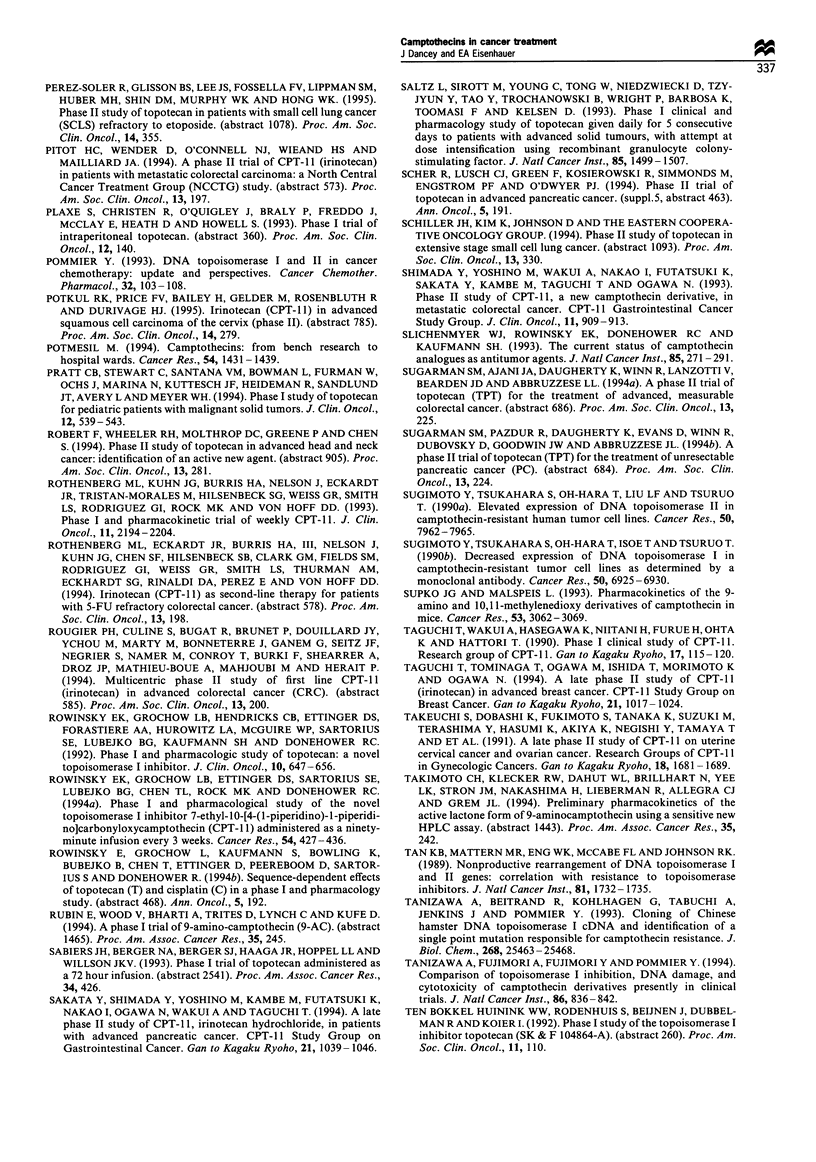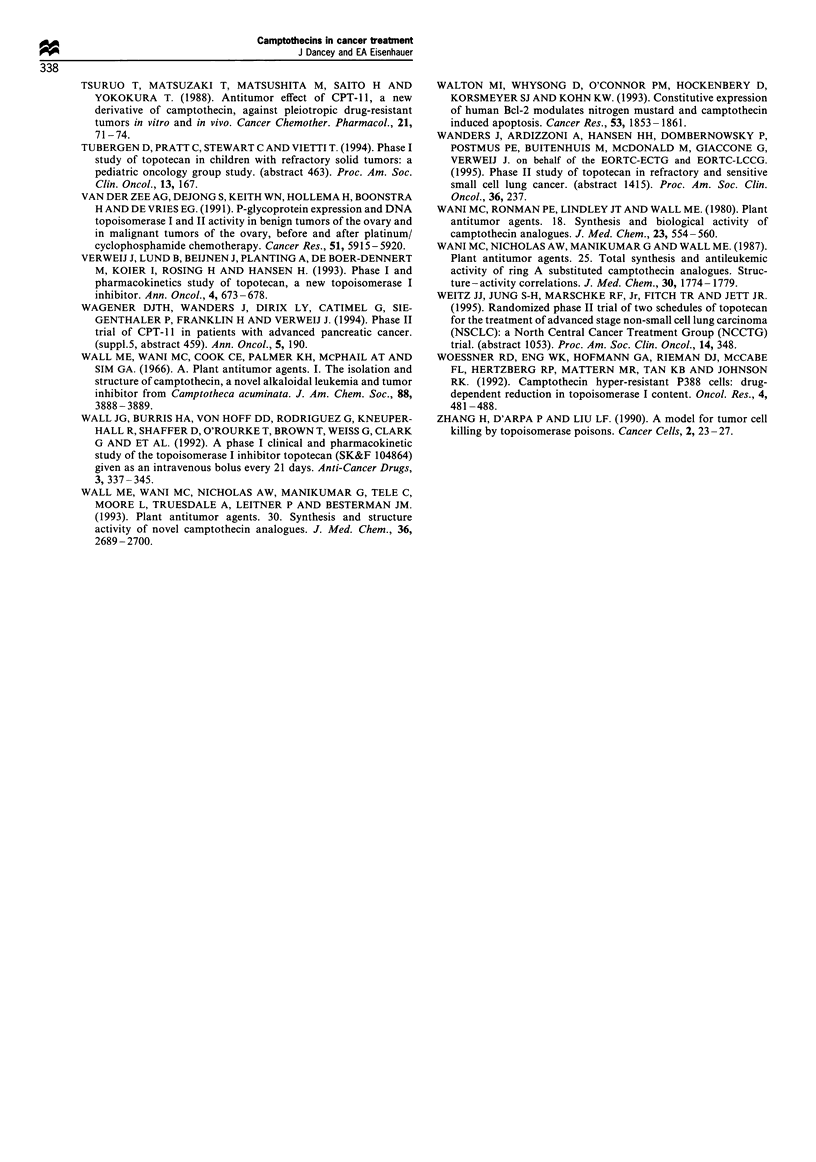# Current perspectives on camptothecins in cancer treatment.

**DOI:** 10.1038/bjc.1996.362

**Published:** 1996-08

**Authors:** J. Dancey, E. A. Eisenhauer

## Abstract

The camptothecins are a new class of chemotherapeutic agents which have a novel mechanism of action targeting the nuclear enzyme topoisomerase I. Knowledge of the structure-activity relationships of the parent compound camptothecin has led to the development of effective soluble analogues with manageable toxicities. Broad anti-tumour activity shown in preclinical studies has been confirmed in phase I/II studies for irinotecan and topotecan. Two other derivatives, 9-aminocamptothecin and GI 147211C, are undergoing phase I and early phase II evaluation. Although camptothecin is a plant extract, it and most of its derivatives are not affected by the classic P-gpMDR1 mechanism of resistance which may allow the development of novel combination chemotherapeutic regimens. Important areas of future endeavour will include the development of rational combination regimens and the pursuit of randomised trials. Based on single agent data, colorectal cancer and non-small-cell lung cancer should be the focus for future irinotecan studies. Small-cell lung cancer and ovarian carcinoma are logical tumour types to pursue with topotecan. Both 9-aminocamptothecin and GI 147211C are too early in their clinical evaluation to make recommendations about their future roles. Finally, the unfolding story of camptothecin analogue development will give important insights into the predictive value of preclinical observations on relative efficacy, schedule dependency, combination strategies and resistance mechanisms which have helped determine the strategies for clinical evaluation of these agents.


British Journal of Cancer (1996) 74, 327-338
© 1996 Stockton Press All rights reserved 0007-0920/96 $12.00

EDITORIAL

### Current perspectives on camptothecins in cancer treatment

J Dancey and EA Eisenhauer

*National Cancer Institute (Canada) Clinical Trials Group, Queen's University, 82-84 Barrie Street, Kingston, Ontario, Canada K76 3N6.*

**Summary** The camptothecins are a new class of chemotherapeutic agents which have a novel mechanism of action targeting the nuclear enzyme topoisomerase I. Knowledge of the structure-activity relationships of the parent compound camptothecin has led to the development of effective soluble analogues with manageable toxicities. Broad anti-tumour activity shown in preclinical studies has been confirmed in phase I/II studies for irinotecan and topotecan. Two other derivatives, 9-aminocamptothecin and GI 147211C, are undergoing phase I and early phase II evaluation. Although camptothecin is a plant extract, it and most of its derivatives are not affected by the classic P-gp$^{MDR1}$ mechanism of resistance which may allow the development of novel combination chemotherapeutic regimens. Important areas of future endeavour will include the development of rational combination regimens and the pursuit of randomised trials. Based on single agent data, colorectal cancer and non-small-cell lung cancer should be the focus for future irinotecan studies. Small-cell lung cancer and ovarian carcinoma are logical tumour types to pursue with topotecan. Both 9-aminocamptothecin and GI 147211C are too early in their clinical evaluation to make recommendations about their future roles. Finally, the unfolding story of camptothecin analogue development will give important insights into the predictive value of preclinical observations on relative efficacy, schedule dependency, combination strategies and resistance mechanisms which have helped determine the strategies for clinical evaluation of these agents.

**Keywords:** camptothecin; irinotecan; 9-aminocamptothecin; topoisomerase I inhibitor; topotecan

More than 30 years ago, an extract from the Chinese tree *Camptotheca acuminata* was found to have anti-tumour activity in experimental systems (Wall *et al.*, 1966). The active compound, camptothecin, being insoluble in aqueous solution, was modified and its water-soluble sodium salt was evaluated in clinical studies in the late 1960s and early 1970s. Leucopenia and thrombocytopenia were dose-limiting toxicities (Muggia *et al.*, 1972). Despite promising anti-tumour activity in phase I studies, results in phase II trials in patients with gastrointestinal malignancies (Moertel *et al.*, 1972) and melanoma (Gottlieb, 1972) indicated the drug was ineffective and highly toxic. Myelosuppression, vomiting, diarrhoea and sterile haemorrhagic cystitis were often severe and, as a result, further clinical testing of camptothecin ceased.

Several developments in the late 1980s renewed interest in camptothecin: topoisomerase I was identified as the cellular target of the drug (Hsiang *et al.*, 1988); the structure-activity relationship was determined for camptothecin (Jaxel *et al.*, 1989), leading to the development of effective, water-soluble synthetic and semisynthetic derivatives (Wall *et al.*, 1993); and topoisomerase I levels were found to be higher in some tumour tissues compared with the normal tissue counterpart (Giovanella *et al.*, 1989; Van der Zee *et al.*, 1991). Currently camptothecin and four analogues: topotecan, irinotecan (CPT-11), 9-aminocamptothecin and GI 147211C (GG211) are undergoing clinical evaluation. During the past 25 years, knowledge of topoisomerase biochemistry, genetics, molecular biology and interaction with inhibitors has increased exponentially and these will be reviewed with the results of preclinical and clinical evaluations of camptothecin and its derivatives.

### DNA topoisomerase I

Topoisomerases are nuclear enzymes that modulate the three-dimensional structure of DNA by inducing transient breaks that allow unwinding of supercoiled DNA (reviewed in Pommier, 1993). Topoisomerase I is a 100 000 kDa protein which relaxes positive and negative supercoils of DNA arising during DNA and RNA synthesis by making transient single-stranded breaks in DNA (Champoux, 1976). Intense research has clarified the camptothecin-topoisomerase I-DNA interaction. The drug binds to and stabilises the topoisomerase I enzyme-DNA cleavable complex after DNA cleavage preventing resealing of DNA and causing an accumulation of cleavable complexes (Hsiang *et al.*, 1988, 1989). The subsequent interaction between the advancing replication fork of DNA and the drug-stabilised cleavable complex results in an arrest of DNA replication with formation of double-strand breaks. These in turn activate endonucleases, triggering further DNA fragmentation and ultimately cell death (Zhang *et al.*, 1990). Thus, cytotoxicity is dependent on the expression of topoisomerase I and on DNA replication. Compared with the levels of the enzyme in normal tissues, a significant increase of topoisomerase I has been detected in surgical specimens of colon adenocarcinoma, ovarian and oesophageal carcinoma, in cultures of non-Hodgkin's lymphoma and leukaemia cells and in xenograft lines of human colon adenocarcinoma, carcinoma of the stomach, breast, lung and malignant melanoma (Potmesil, 1994). Cell lines which have high levels of enzyme are hypersensitive to camptothecin-induced cytotoxicity (Madden, 1992). Conversely, cell lines resistant to camptothecin may contain qualitatively or quantitatively altered forms of the target enzyme (Pommier, 1993). Although topoisomerase I is expressed throughout the cell cycle, cells in S-phase are 1000 times more sensitive than cells in $G_1$ or $G_2$-phase to the cytotoxicity of camptothecins reflecting the need for DNA replication for drug efficacy (Del Bino *et al.*, 1991). Although much is known, our understanding of the mechanism of activity of these agents might be incomplete. These agents are active in human tumour xenografts that typically have low S-phase fractions and studies have shown that the fraction of cells killed by a brief exposure to camptothecin is sometimes larger than the S-phase fraction of the cell population, thus other cellular effects of camptothecins may be linked to cytotoxicity (O'Connor *et al.*, 1991).

Structure-activity experiments have defined the features of the molecule critical for cytotoxicity. Camptothecin has a heterocyclic five-ring structure with a lactone moiety and an

Correspondence: E Eisenhauer
Received 27 February, 1996

S-hydroxyl moiety on ring E (Figure 1). Camptothecin lactone exists in a pH-dependent equilibrium with an open ring carboxylate form. At physiological pH 90% of the drug exists as carboxylate. Both the pentacyclic ring structure of camptothecin, and the lactone and hydroxyl moieties are required for cytotoxicity as molecules with fewer than five rings, or bearing either a 20(R) hydroxyl or the open ring carboxylate are biologically inactive (Hertzberg *et al.*, 1989*a*,*b*). Substitutions at positions 9 or 10 by amino or hydroxyl groups lead to compounds with equal or greater *in vivo* activity than the parent compound (Wani *et al.*, 1980, 1987). Knowledge of these features has led to the development of analogues of camptothecin which are both water soluble and effective. Four analogues are now undergoing clinical evaluation: irinotecan, topotecan, 9-aminocamptothecin and GI 147211 (GG211).

### Irinotecan (CPT-11)

### *Preclinical studies*

The first of the water-soluble analogues is irinotecan (CPT-11) or 7-ethyl-10-(4-[1-piperidino]-1-piperidino)methyl-10-hydroxycamptothecin. Irinotecan, a prodrug with limited activity, is converted in plasma by de-esterification into SN-38 which has 1000 times the potency of the parent compound (Kawato *et al.*, 1991*a*).

Irinotecan is active against a diverse array of tumour cell lines *in vitro* and *in vivo*. The SN-38 metabolite is a more effective inhibitor of topoisomerase I and more cytotoxic toward HT-29 human colon carcinoma cells in culture than camptothecin, 9-aminocamptothecin and topotecan (Tanizawa *et al.*, 1994). Irinotecan, when given by intraperitoneal, intravenous or oral routes, showed substantial activity against a broad spectrum of mouse and human tumour xenografts including human cancer xenograft lines resistant to topotecan, vincristine or melphalan (Kunimoto *et al.*, 1987). Interestingly, sensitivities of some tumour cell lines to irinotecan were independent of their ability to produce SN-38 suggesting that cytotoxicity is not solely dependent on the production of the metabolite (Kawato *et al.*, 1991*b*). Unlike 9-aminocamptothecin and topotecan, the efficacy of irinotecan was not substantially influenced by administration schedule in preclinical studies (Furuta, 1990).

### *Clinical studies of irinotecan*

Clinical evaluation of irinotecan is well advanced. Phase I trials (Table I) were conducted in Japan and more recently in the United States and Europe on several schedules: 30 min infusion every week and every 3 weeks; 30–90 min infusion daily for 3 days every 3 weeks; 90 min infusion every week and 3 weeks; and 120 h continuous intravenous infusion every 3–4 weeks. Dose-limiting toxicities were somewhat dependent on the treatment schedule. Dose-limiting leucopenia, neutropenia and diarrhoea were prominent in single-dose regimens, while gastrointestinal toxicities prevailed with c.i.v. schedules. Diarrhoea is the most significant gastrointestinal toxicity and may occur early or late following treatment. The early syndrome begins during or shortly after the infusion of irinotecan and is often associated with flushing, sweating, nausea, vomiting and abdominal cramps. Both inhibition of acetylcholinesterase at muscarinic receptors (Kawato *et al.*, 1993) and stimulation of the nicotinic receptors in autonomic ganglion cells (Gandia *et al.*, 1993) have been postulated as mechanisms. It can be managed by the administration of diphenhydramine or atropine with a serotonin antagonist such as ondansetron. Late diarrhoea begins 1–3 weeks after treatment and may last 5–7 days. Its occurrence is unpredictable and it may be severe. It is refractory to most antidiarrhoeal agents including opiates, atropine and octreotide but it may respond to high-dose loperamide with or without the enkephalinase inhibitor acetorphan (Hagipantelli *et al.*, 1995). In one small study, early aggressive treatment with loperamide 2 mg every 2 h (24 mg per 24 h) until 12 h without a bowel movement reduced the incidence of severe diarrhoea to 6% (Abigerges *et al.*, 1994). Other toxic effects of irinotecan included thrombocytopenia, eosinophilia, anaemia, alopecia, fatigue, transient elevation of liver function tests, rash and mucositis. Rarely, cases of

**Figure 1** CPT, camptothecin; TPT, topotecan; 9-AC, 9-aminocamptothecin; CPT-11, irinotecan; GG211, GI147211C.

interstitial pneumonitis have occurred in previously treated patients with lung cancer.

The pharmacokinetics of irinotecan are complex. In plasma, carboxylesterases rapidly convert the prodrug into SN-38 and both irinotecan and SN-38 are converted by pH-dependent hydrolysis from lactone to carboxylate forms. Concentrations of the irinotecan lactone in plasma is almost two orders of magnitude higher than that of SN-38 and both lactone forms represent 44% and 50% of the total drug and metabolite detectable in plasma (Rowinsky *et al.*, 1994*a*). Peak plasma levels and AUC of irinotecan correlate well with dose (De Forni *et al.*, 1994). The AUC of SN-38 correlates with the AUC but not the dose of irinotecan. The reported terminal half-life of irinotecan is 5.2–9.3 h, and the mean residence times for it and SN-38 are 9.1 and 10.0 h. Hydrolysis of irinotecan and SN-38 lactone is less than for topotecan and 9-aminocamptothecin with 33–66% remaining intact at 24 h after infusion of irinotecan. The maintenance of biologically relevant concentrations of SN-38 for long durations may explain the observation that anti-tumour efficacy of irinotecan is not schedule dependent. There is significant interpatient variability in the conversion of irinotecan to SN-38, implying that dose increases may not lead to proportional increases in cytotoxicity.

Biliary and urinary excretion are both important routes of elimination. In humans $37 \pm 4\%$ of the drug is detected in urine in 48 h (Rowinsky, 1994*a*). Both irinotecan and SN-38 undergo glucuronic acid conjugation and are eliminated in bile (Narita *et al.*, 1993; Gupta *et al.*, 1994). $\beta$-Glucuronidase of intestinal microflora can cleave the glucuronide and free intestinal SN-38 which may play a role in producing the late diarrhoea. Indirect estimates of biliary concentration of SN-38 and its glucuronide have shown good correlation between the concentration of SN-38 and the occurrence of late diarrhoea (Araki *et al.*, 1993).

In single agent phase II trials (Table II), irinotecan was active against a wide range of carcinomas and lymphomas. Activity was observed using schedules of 100–150 mg $m^{-2}$ $week^{-1}$ and 350 mg $m^{-2}$ every 3 weeks. The response rates appear to favour weekly administration but direct comparisons between the two schedules have not been made. Of particular interest are the results of five trials in patients with metastatic colorectal cancer where response rates have ranged from 14% to 32%. Similar levels of activity have been seen in untreated colorectal patients, patients previously treated with 5-FU and patients who were treated after progressing on 5-FU (Bugat *et al.*, 1995). Response rates of 34% and 36% were observed in untreated patients with non-small-cell lung cancer. Complete and partial remissions were seen in 24% of ovarian cancer and 23% of breast cancer patients who had received prior chemotherapy. Results of three studies in patients with cervical cancer have been mixed, perhaps reflecting different schedules and patient populations. On the weekly schedule response rates of 24–27% were observed in patients previously treated with cisplatin but 0% in patients who were refractory to cisplatin. A response rate of 15% was observed in chemotherapy-naive patients on the 3 weekly schedule but 24% in the subset of 21 patients who had measurable disease outside previously irradiated fields. Partial responses were seen in 23% of patients with advanced gastric cancer, 40% of patients with small-cell lung cancer and in 39% with non-Hodgkin's lymphoma. Thus, irinotecan has impressive activity in many malignancies particularly colorectal carcinoma, non-small-cell lung cancer and cervical carcinoma. Toxic effects are usually manageable but late diarrhoea may be severe despite maximal medical therapy. Because of this it may be challenging to combine this drug with other cytotoxic agents particularly those with similar toxicities. A direct comparison of the weekly and 3 weekly schedule is an obvious question to be addressed in a comparative trial.

### *Future directions*

Future study of irinotecan will be likely to fall into three major areas: the pursuit of effective (preferably mechanism-based) methods of overcoming the late diarrhoea, the development of rational, safe combination regimens (see section on Combination treatment) and the randomised comparison of irinotecan-based regimens with standard therapy. The tumour types which ought to be the focus of the initial group of comparative trials include both colorectal cancer and non-small-cell lung cancer based on the single agent data in these diseases.

Secondary areas of endeavour which merit evaluation include further phase II testing, particularly in those tumour types with supportive preclinical data such as sarcoma and glioma, and the development of a better understanding of the pharmacokinetic/dynamic relationship of the drug to toxic and efficacy outcomes.

### Topotecan

### *Preclinical studies*

Topotecan (9-(dimethylamino)methyl-10-hydroxycamptothecin) is a camptothecin derivative having aqueous solubility conferred by the charged amino group on the 9-substituent. When tested against a variety of transplantable mouse and human tumours, topotecan demonstrated anti-tumour

**Table I** Phase I studies of irinotecan (CPT-11)

| *Reference* | *Schedule* | *MTD (mg $m^{-2}$ $day^{-1}$)* | *Phase II (mg $m^{-2}$)* | *Limiting toxicity* | *Responses* |
|---|---|---|---|---|---|
| Taguchi *et al.* (1990) | 60 min i.v. q28d | 250 | 200 | Neutropenia | Not reported |
| Rowinsky *et al.* (1994*a*) | 90 min i.v. q21d | 290 | 240 | Neutropenia, nausea, vomiting | 1 PR rectal<br>1 PR cervix<br>1 PR RCC |
| Abigerges *et al.* (1994) | 30 min i.v. q21d | >600 | ND | ND | 4 PR colon<br>1 PR cervix |
| De Forni *et al.* (1994) | 30 min i.v. qwk | 145 | 115 | Neutropenia | 1 PR oesophagus |
| Negoro *et al.* (1991*a*) | 90 min i.v. qwk | 150 | 100 | Leucopenia, diarrhoea | 2 PR NSCLC |
| Rothenberg *et al.* (1993) | 90 min i.v. qwkx4 q6wk | 180 | 150 | Diarrhoea | 2 PR colon |
| Lestingi *et al.* (1995) | 90 min i.v. qwkx4 q6wk | 175 | 145* | Neutropenia, diarrhoea | 1 PR gastric |
| Ohe *et al.* (1992) | 120 h c.i.v. q3–4wk | 40 | 30 | Diarrhoea | Not reported |
| Clavel *et al.* (1992) | 30–90 min i.v. × 3d q21d | 145 | 100 | Neutropenia | 1 PR mesothelioma<br>1 PR breast |

MTD, maximum tolerated dose; PR, partial response; ND, not determined; RCC, renal cell carcinoma; NSCLC, non-small-cell ling carcinoma; c.i.v., continuous intravenous infusion; *G-CSF and aggressive antidiarrhoeal support.

activity when administered intravenously, intraperitoneally, subcutaneously or orally (McCabe, 1994). Preclinical testing indicated that, in topotecan-sensitive tumours, longer exposure to the drug increased the magnitude of response (Burris *et al.*, 1992; Friedman *et al.*, 1994). This could indicate that, in the clinic, more prolonged schedules of administration might be superior to short infusions.

*Clinical studies of topotecan*

Based on the results or preclinical screening, topotecan entered phase I studies in the United States and Europe (Table III). Short, intermediate and prolonged infusion schedules have been studied including: single intravenous injection every 21 days; 30 min infusion on 5 consecutive days every 21–28 days; 24, 72, 96 and 120 h continuous intravenous infusions every 21–28 days; and a 21 day continuous intravenous infusion every 28 days. In all studies myelosuppression was dose-limiting although the pattern of myelosuppression varied with the method of administration. Intermittent bolus and short infusion schedules resulted in non-cumulative neutropenia as the predominant toxicity, whereas prolonged continuous infusions were followed by neutropenia, thrombocytopenia and anaemia. Non-haematological toxic effects were generally mild and included alopecia, nausea, vomiting, diarrhoea, elevations in hepatic enzymes, mucositis, skin rash and fatigue.

Pharmacokinetic studies of topotecan show the drug is rapidly hydrolysed in plasma to the open-ring form following intravenous administration (Rowinsky *et al.*, 1992). The plasma clearance is biexponential with a terminal half-life of 3 h. Renal elimination is important as 40% of the drug is excreted in the urine within the first 24 h of treatment. There is a good correlation between the AUC of total topotecan (lactone plus hydroxyacid) and the grade of neutropenia in patients with normal or impaired renal or hepatic function (Grochow *et al.*, 1994). Patients with reduced creatinine clearance require dose adjustment as they are at increased risk of toxicity from topotecan. However, hyperbilirubinaemia does not alter topotecan disposition or toxicity and no dose adjustment is required in patients with serum bilirubin as high as 170 $\mu$mol $l^{-1}$.

Although the dose-limiting toxicity of topotecan was neutropenia, attempts to improve dose intensity on the daily × 5 schedule by using haematopoietic growth factors were not successful (Murphy *et al.*, 1992; Rowinsky *et al.*, 1992; Janik *et al.*, 1993).

Since several anti-tumour responses were seen in the daily × 5 phase I trial, this schedule was selected for phase II evaluation. Following this decision, the 21-day c.i.v. phase I trial was completed and also appeared active, thus a limited number of phase II trials have been initiated with the more prolonged schedule.

Because of its broad activity in phase I studies, phase II studies of topotecan were initiated for many different tumour types (Table IV). In a randomised phase II study comparing the daily × 5 day schedule to 72 h c.i.v. schedule in untreated patients with advanced NSCLC, response rate, median time to progression and median survival favoured the daily for 5 days schedule (Weitz *et al.*, 1995). In untreated colorectal cancer an 8% response rate was seen using the 21-day c.i.v. schedule. This response rate was similar to that observed in colorectal carcinoma with the daily × 5 day schedule, but the c.i.v. administration was associated with significant cumulative myelosuppression and pronounced anaemia (Creemer *et al.*, 1995). Activity in other tumour types on the daily × 5 schedule included response rates of 40% and 21% in untreated and previously treated SCLC respectively, 33% in breast cancer and 27% in head and neck cancer. In two trials involving heavily pretreated patients

**Table II** Irinotecan (CPT-11) phase II trials

| *Reference* | *Tumour site* | *Evaluable patients* | *Prior Rx* | *Dose schedule ($mg\ m^{-2}\ dose^{-1}$)* | *CR* | *Response PR* | *%* |
|---|---|---|---|---|---|---|---|
| Ohno *et al.* (1990)[a] | Lymphoma | 66 | All | 40 daily × 3d qwk | 9 | 17 | 39 |
| Ota *et al.* (1994) | Leukaemia | 41 | All | 15–20 b.i.d. × 7d q3–4wk | 0 | 2 | 5 |
| Sakata *et al.* (1994) | Pancreas | 35 | Some | 100 qwk<br>150 q2wk | 0 | 4 | 11 |
| Wagener *et al.* (1994) | Pancreas | 18 | None | 350 q3wk | 0 | 3 | 15 |
| Takeuchi *et al.* (1991)[a] | Ovary | 55 | 52 CDDP<br>4 XRT | 100 qwk | 0 | 13 | 24 |
| | Cervix | 55 | 30 CDDP<br>52 XRT | 150 q2wk | 5 | 8 | 24 |
| Kavanagh *et al.* (1994) | Cervix | 11 | All CDDP | 150 qwk × 4wks<br>q6wks | 1 | 2 | 27 |
| Potkul *et al.* (1995) | Cervix | 14 | All CDDP<br>resistant | 125 qwk × 4wk q6wk | 0 | 0 | 0 |
| Chevallier *et al.* (1995) | Cervix | 34 | No CT | 350 q3wks | 1 | 4 | 15[b] |
| Douillard *et al.* (1995) | NSCLC | 11 | No CT | 350 q3ks | 0 | 4 | 36 |
| Negoro *et al.* (1991*b*)[a] | NSCLC | 67 | None | 100 qwk | 0 | 23 | 34 |
| Masuda *et al.* (1992) | NSCLC | 26 | All | 100 qwk | 0 | 0 | 0 |
| | SCLC | 8 | None | 100 qwk | 0 | 4 | 50 |
| | SCLC | 27 | All | 100 qwk | 2 | 7 | 33 |
| Bonneterre *et al.* (1993) | Breast | 12 | All | 350 q3wk | 1 | 0 | 8 |
| Taguchi *et al.* (1994)[a] | Breast | 65 | 46 CT | 100 qwk | 1 | 14 | 23 |
| Shimada *et al.* (1993) | Colon | 63 | 51 CT | 100 qwk<br>150 q2wk | 0 | 17 | 27 |
| Pitot *et al.* (1994) | Colon | 34 | 21 CT | 125 weekly × 4<br>q6wk | 0<br>0 | 5<br>2 | 24<br>15 |
| Rothenberg *et al.* (1994) | Colon | 44 | All | 125–150 weekly × 4 q6wk | 1 | 10 | 25 |
| Bugat *et al.* (1994) | Colon | 85 | All | 350 q3wk | 2 | 10 | 14 |
| Rougier *et al.* (1994) | Colon | 35 | None | 350 q3wk | 0 | 7 | 20 |
| Conti *et al.* (1994) | Colon | 19 | None | 125 weekly × 4<br>q6wk | 0 | 6 | 32 |
| Futatsuki *et al.* (1994) | Gastric | 60 | 45 CT | 100 qwk<br>150 q2wk | 0 | 14 | 23 |

[a]Studies with early and late results. [b]1 CR and 3 PR (24%) in 21 patients with measurable disease outside previously irradiated areas.

with carcinoma of the ovary, response rates of 14% and 25% were seen. In both studies, the majority of non-responding patients had prolonged disease stabilisation, an observation reminiscent of results with taxoids in platinum-treated patients. Minimal activity was observed against carcinomas of the pancreas, prostate, kidney, NSCLC, melanoma, mesothelioma, soft-tissue sarcoma and glioma.

*Future directions*

Based on its promising single agent phase II results, further studies of topotecan in combination with other effective cytotoxic agents are warranted in SCLC, breast cancer, head and neck cancer and ovarian cancer (see section on Combination treatment). Small-cell lung cancer and ovarian

**Table III** Topotecan phase I studies

| *Reference* | *Schedule* | *MTD mg $m^{-2}$ $dose^{-1}$* | *Phase II mg $m^{-2}$ $dose^{-1}$* | *Limiting toxicity* | *Objective responses* |
|---|---|---|---|---|---|
| Wall *et al.* (1992) | 30 min i.v. q21d | 22.5 | 20 | Neutropenia | None |
| Hasegawa *et al.* (1993) | 30 min i.v. q21d | 22.5 | 20 | Leucopenia | Not reported |
| Blaney *et al.* (1993)[a] | 24 h c.i.v. q21d | 7.5[b] | 5.5 | Leucopenia, thrombocytopenia | None |
| Abbruzzese *et al.* (1993) | 24 h c.i.v. q21d | 12.5 | 10[c] | Neutropenia | None |
| ten Bokkel Huinink *et al.* (1992) | 24 h c.i.v. q21d | 10.5 | 8.4 | Neutropenia, thrombocytopenia | None |
| Haas *et al.* (1994) | 24 h c.i.v. qwk | 1.75 | 1.5 | Neutropenia | 1 PR colon |
| Pratt *et al.* (1994)[a] | 72 h c.i.v. q21d G-CSF | 3.9 | 3.0 | Neutropenia, thrombocytopenia | 1 CR neuroblastoma |
| Sabiers *et al.* (1993) | 72 h c.i.v. qwk<br>72 h c.i.v. q2wk | 2<br>2.6 | 2 | Myelotoxicity | Not reported |
| Burris *et al.* (1994) | 72 h c.i.v. q21d<br>120 h c.i.v. q21d | 4.8<br>3.4 | 4.8[d] | Neutropenia, thrombocytopenia | None |
| Kantarjian *et al.* (1993)[e] | 120 h c.i.v. q21–28d | 11.8 | 10 | Mucositis | 2 CR AML<br>1 CR CML-BC<br>2 PR AML |
| Rowinsky *et al.* (1992) | 30 min i.v. × 5d q21d | 2 | 1.5 | Neutropenia | 1 CR NSCLC<br>1 CR ovary<br>2 PR NSCLC |
| Saltz *et al.* (1993) | 30 min i.v. × 5d q28d | 1.75 | 1.5 | Neutropenia | 1 PR oesophagus<br>1 PR CUP |
| Verweij *et al.* (1993) | 30 min i.v. × 5d q21d | 1.5 | 1.5 | Leucopenia | 1 PR SCLC<br>1 PR NSCLC<br>1 PR pancreas |
| Tubergen *et al.* (1994) | 30 min i.v. × 5d q21d G-CSF | 2.4 | 2.0 | Neutropenia, thrombocytopenia | None |
| Hochster *et al.* (1994) | 21d c.i.v. q28d | 14.7 | 11.3 | Neutropenia, thrombocytopenia | 2 PR ovary<br>1 PR NSCLC<br>1 PR breast |
| Plaxe *et al.* (1993) | 24 h c.i.p. q28d[f] | 4 | 3 | Neutropenia | 5 reduction of ascites |

[a]Paediatric patients; [b]for c.i.v. schedules dose cited is total dose over the total time of infusion; [c]dose for previously untreated patients without G-CSF; MTD, 15 mg $m^{-2}$ with G-CSF; [d]recommended schedule for higher dose intensity; [e]all leukaemia patients; [f]c.i.p, continuous intraperitoneal infusion.

**Table IV** Topotecan phase II studies

| *Reference* | *Tumour site* | *Evaluable patients* | *Prior Rx* | *Dose schedule mg $m^{-2}$ $dose^{-1}$* | *Response CR* | *PR* | *%* |
|---|---|---|---|---|---|---|---|
| Giantonio *et al.* (1993) | Prostate | 28 | Hormones | 1.5 × 5 days q21d | 0 | 1 | 5 |
| Ilson *et al.* (1993) | Renal cell | 15 | No CT | 1.5 × 5 days q21d | 0 | 0 | 0 |
| Kudelka *et al.* (1993) | Ovary | 28 | All CT | 1.5 × 5 days q21d | 0 | 4 | 14 |
| Armstrong *et al.* (1995) | Ovary | 16 | All CDDP refractory | 1.5 × 5 days q21d | 1 | 3 | 25 |
| Chang *et al.* (1995) | Breast | 15 | 0–1 regimens | 1.5 × 5 days q21d | 0 | 5 | 33 |
| Eisenhauer *et al.* (1994) | Sarcoma | 29 | None | 1.5 × 5 days q21d | 0 | 3 | 10 |
| Eisenhauer *et al.* (1994) | Glioma | 31 | CT 12<br>XRT 24 | 1.5 × 5 days q21d | 1 | 1 | 7 |
| Creemers *et al.* (1994) | Colon | 28 | None | 1.5 × 5 days q21d | 0 | 2 | 7 |
| Sugarman *et al.* (1994*a*) | Colon | 19 | Unknown | 1.5 × 5 days q21d | 0 | 0 | 0 |
| Creemers *et al.* (1995) | Colon | 16 | None | 0.6 c.i.v. × 21d q 28d | 1 | 0 | 6 |
| Robert *et al.* (1994) | Head and neck | 15 | No CT | 1.5 × 5 days q21d | 0 | 4 | 27 |
| Schiller *et al.* (1994)<br>Hutson *et al.* (1995) | SCLC | 35 | No CT | 2.0 × 5 days q 21d G-CSF | 0 | 14 | 40 |
| Wanders *et al.* (1995) | SCLC | 57 | All CT | 1.5 × 5 days q21d | 2 | 7 | 21 |
| Perez-Soler *et al.* (1995) | SCLC | 25 | All refractory to etoposide | 1.25 × 5 days q21d | 0 | 3 | 12 |
| Weitz *et al.* (1995) | NSCLC | 38<br>36 | No CT | 1.5 × 5 days q21d<br>1.3 c.i.v. × 3 d q 28d | 0<br>0 | 5<br>2 | 18<br>8 |
| Perez-Soler *et al.* (1994) | NSCLC | 37 | None | 1.5 × 5 days q21d | 0 | 5 | 14 |
| Lynch *et al.* (1994) | NSCLC | 20 | None | 2.0 × 5 days q21d | 0 | 0 | 0 |
| Maksymiuk *et al.* (1995) | Mesothelioma | 22 | None | 1.5 × 5 days q21d | 0 | 0 | 0 |
| Scher *et al.* (1994) | Pancreas | 34 | No CT | 1.5 × 5 days q21d | 0 | 4 | 12 |
| Sugarman *et al.* (1994*b*) | Pancreas | 15 | None | 1.5 × 5 days q21d | 0 | 0 | 0 |

CT, chemotherapy; XRT, radiation therapy; CR, complete response; PR partial response.

cancer have already been identified as tumour types for randomised studies. In the former, topotecan is being evaluated in a front-line setting, while in ovarian cancer, a randomised comparison with paclitaxel in platinum pre-treated patients has been completed, although results are not yet available. The findings of this ovarian trial and that of a phase II study of topotecan in paclitaxel failures will be important in determining if front-line regimens incorporating this new drug should be developed further.

At the present time the daily for 5 days schedule appears to offer the best balance of efficacy and toxicity compared with 72 h or 21-day c.i.v. schedules, despite preclinical data favouring prolonged drug exposures. However, the optimal schedule of administration of topotecan may not yet be defined and several trials evaluating the activity of the 21-day infusion are ongoing. The results of these studies may lead to an interest in chronic oral dosing strategies.

Finally, the evidence of a relationship between total topotecan AUC and neutropenia (Grochow *et al.*, 1994) together with recent publication of a limited sampling model for determining topotecan AUC (Minami *et al.*, 1996) should lead to prospective studies assessing the pharmacokinetic/dynamic behaviour of this agent.

### 9-Aminocamptothecin

Among the many semisynthetic or totally synthetic camptothecin analogues screened, 9-aminocamptothecin was selected for advanced testing and clinical development primarily because of its ability to induce complete remissions in mice bearing human colonic adenocarcinoma and malignant melanoma cell lines known to be resistant to standard chemotherapeutic agents (Giovanella *et al.*, 1989, 1991; Pantazis *et al.*, 1992). Like topotecan, pharmacokinetic and efficacy studies of 9-aminocamptothecin suggested that maintaining the lactone plasma concentration above a threshold level for a prolonged period was required for optimal therapeutic effect (Supko *et al.*, 1993).

The innate aqueous insolubility of 9-aminocamptothecin resulted in difficulty devising a suitable formulation and delayed initiation of phase I studies. Two studies of 9-aminocamptothecin, formulated in polyethylene glycol 400, phosphoric acid and dimethylacetamide, have been completed. In both, the drug was given as a 72 h continuous intravenous infusion either every 2 weeks or every 3 weeks in patients with advanced solid tumours (Dahut *et al.*, 1994; Rubin *et al.*, 1994). The 72 h infusion was selected to try to achieve the prolonged drug concentrations above a 'threshold' level known to be of importance to anti-tumour effect in animal model systems. Preliminary reports indicate the drug formation and schedule were well tolerated. Leucopenia was dose-limiting in both trials. In one study the maximum tolerated dose (MTD) was 59$\mu$g $m^{-2}$ $h^{-1}$ and the dose was escalated to 74 $\mu$g $m^{-2}$ $h^{-1}$ with granulocyte colony-stimulating factor (G-CSF). With the highest doses, grade 3 thrombocytopenia as well as nausea/vomiting (controlled with antiemetics), total alopecia, stomatitis and, infrequently, diarrhoea were seen. In the 19 patients evaluated in this phase I study of 9-AC every 2 weeks, there were no objective responses; minimal responses were evident in patients with colon, lung and gastric carcinomas (Dahut *et al.*, 1994).

Preliminary pharmacokinetic studies of 9-aminocamptothecin given as a 72 h infusion were done as part of the phase I evaluation (Takimoto *et al.*, 1994). Steady-state plasma concentrations increased linearly from $0.89 \pm 0.63$ nM to $5.6 \pm 0.6$ nM over the dose range of 5 to 59 $\mu$g $m^{-2}$ $h^{-1}$ and total body clearance was $26.5 \pm 8.6$ ml min $m^{-2}$. Non-linear regression analysis demonstrated biphasic pharmacokinetics for 9-aminocamptothecin lactone with a $t_{1/2\alpha}$ of 1.5–2.5 h and a $t_{1/2\beta}$ of 10.7–12.9 h. Mean steady-state plasma levels of 9-aminocamptothecin lactone correlated well with the percentage decrease in granulocyte and leucocyte counts. Phase II testing of 9-aminocamptothecin as a 72 h infusion every 2 weeks is ongoing. Furthermore, clinical testing of a colloid dispersion formulation which improves the aqueous solubility of 9-aminocamptothecin 20-fold is under way. Definitive comments on schedule, formulation and efficacy await the results of these studies.

### GI 147211C (GG211)

GI 147211C (recently renamed GG211) is the synthetic water-soluble camptothecin analogue, 7-(4-methylpiperazinomethylene)-10,11-ethylenedioxy-20(s)-camptothecindihydrochloride. In comparison with topotecan *in vitro*, GI 147211C is a more potent inhibitor of topoisomerase I and has greater cytotoxicity (Kang *et al.*, 1993; Emerson *et al.*, 1995). Anti-tumour activity was assessed in xenograft models and its anti-tumour effect was dose schedule-dependent with a greater reduction in tumour volume achieved by prolonged dosing (2× week for 5 weeks). Concurrent experiments demonstrated that GI 147211C was slightly more effective than topotecan in suppressing tumour growth. Preliminary reports of phase I clinical trials of daily times 5 days and 72 h infusion schedules are available. On the daily times 5 every 21 day schedule, the maximal tolerated dose was 1.75 mg $m^{-2}$ $day^{-1}$ in minimally pretreated patients and 1.2 mg $m^{-2}$ $day^{-1}$ in heavily pretreated patients (Eckardt *et al.*, 1995). Dose-limiting toxicities were neutropenia and thrombocytopenia with no evidence of cumulative toxicity. With the 72 h c.i.v. schedule, both neutropenia and thrombocytopenia were dose-limiting at the maximum tolerated dose in pretreated patients of 2.0 mg $m^{-2}$ $day^{-1}$ (O'Dwyer *et al.*, 1995). Non-haematological toxicities seen with both schedules were mild and included alopecia, anorexia, fatigue, nausea, vomiting, headache and phlebitis. Responses were seen in patients with breast, ovary and colorectal cancer who received the drug on the 72 h c.i.v. schedule.

### Combination treatment

Extensive preclinical investigation has led to specific strategies for combining camptothecins with chemotherapeutic agents and radiation. *In vitro* and, for some drugs, *in vivo* studies show that the efficacy of camptothecins is synergistic or additive when compared sequentially with alkylating agents (cisplatin and cyclophosphamide) (Kano *et al.*, 1992), topoisomerase II inhibitors (doxorubicin, daunorubicin and etoposide) (Del Bino *et al.*, 1992) but antagonistic when combined with the antimetabolite methotrexate. Efficacy of drug combinations depended not only on choice of drug but also on schedule as the administration of camptothecins concurrently with some chemotherapeutic agents leads to antagonistic rather than synergistic effects (Bertrand *et al.*, 1992; Kaufmann, 1991).

The combination of irinotecan and cisplatin was superior to combinations of cisplatin with vindesine or etoposide against human lung adenocarcinoma cell lines (Kuraishi *et al.*, 1992). The scheduling of the drugs was critical for success as sequential administration of camptothecins followed by cisplatin led to synergistic cytotoxicity while concurrent administration led to antagonism. Camptothecins may inhibit topoisomerase I-mediated repair of alkylating agent-induced DNA damage. There is clinical evidence to support these laboratory observations. In a phase I trial, toxicity of topotecan and cisplatin was schedule-dependent. The administration of cisplatin on day 1 followed by topotecan daily for 5 days resulted in greater neutropenia and thrombocytopenia than administration of cisplatin after topotecan (Rowinsky *et al.*, 1994*b*). Studies are underway to determine which regimen possesses superior antineoplastic effect. In a phase I study of irinotecan with cisplatin, the partial remission rate was 54% in patients with NSCLC (Masuda *et al.*, 1992) and in a phase II study of the same drugs in untreated patients with extensive and limited SCLC

the response rates were 79% and 78% respectively. Although these two studies do not answer the question of appropriate timing of drug administration, these results are similar to standard therapies and suggest that the combination is effective (Fujiwara *et al.*, 1994).

Synergy was also seen *in vitro* when camptothecins were administered sequentially but not concurrently with topoisomerase II inhibitors. Pretreatment with irinotecan has been shown to increase in topoisomerase II mRNA in cells and cellular overexpression of topoisomerase II is likely to increase cytotoxicity of topoisomerase II inhibitors (Kim *et al.*, 1992). In a phase I study of topotecan given by continuous infusion on days 1–3 and etoposide given over 2 h on days 7–9, sequential sampling of tumours in five patients was performed. Topoisomerase II levels were markedly increased immediately before etoposide was given on day 7 and levels decreased by day 9 in the tumour cells of one patient who had resolution of malignant ascites (Eckardt *et al.*, 1994). The concurrent administration of irinotecan and etoposide yielded a response rate of 21% in a phase II study of 61 untreated patients with NSCLC (Goto *et al.*, 1995), which was less than that previously reported in two phase II trials of irinotecan alone, suggesting that the *in vitro* data on scheduling is clinically relevant.

Scheduling effects were also seen when camptothecins were combined with radiation in tissue culture cell lines. Synergy was seen only when the drugs were administered shortly after irradiation suggesting low-dose radiation triggers cells to enter S-phase rendering them sensitive to the cytotoxic effects of camptothecins (Mattern *et al.*, 1991; Kim *et al.*, 1992). The clinical relevance of the synergistic effects of camptothecins with radiation has not been determined.

### Drug resistance and camptothecins

The development of cellular resistance to chemotherapeutic agents is an important cause of treatment failure in cancer patients. In the laboratory, at least three well-defined mechanisms of resistance to topoisomerase I inhibitors have been described: alteration of topoisomerase I structure or function; P-glycoprotein (P-gp)-mediated resistance; and, for irinotecan, reduction of conversion of the prodrug to its active metabolite.

Qualitative and quantitative alterations of topoisomerase I are the most significant phenomena causing resistance to camptothecins. In several normal and malignant tissue culture lines relative resistance, measured as the increase in the dose of campotothecin required to produce a given level of survival compared with parental cells, was between 2- and 350-fold (Andoh *et al.*, 1987; Tanizawa *et al.*, 1993). Point mutations (Benedetti *et al.*, 1993), deletions (Sugimoto *et al.*, 1990*b*) and rearrangements (Tan *et al.*, 1989) in the topoisomerase I gene have been reported and may be associated with decreased topoisomerase I levels or activity. The mutations were contained in well-conserved regions of topoisomerase I gene and the domains around the mutations were likely to be critical for enzyme activity and interaction with camptothecins. Deletions and rearrangements may lead to structural and functional alterations of the enzyme and can be accompanied by reduced transcription and enzyme production. Intriguingly, some cell lines which had alterations in topoisomerase I levels and activity were more sensitive to the effects of radiation and topoisomerase II inhibitors (Sugimoto *et al.*, 1990*a*). Preliminary experiments indicated a pattern of cross-resistance among available camptothecins; however, cross-resistance was not absolute as some cell lines resistant to topotecan were sensitive to the cytotoxic effects of irinotecan (Houghton *et al.*, 1993).

Unlike water-insoluble camptothecin and 9-aminocamptothecin, water-soluble derivatives topotecan and irinotecan may be substrates for P-glycoprotein (Chen *et al.*, 1991). Both drugs show reduced cytotoxicity measured by $IC_{50}$ values against $MDR_1$ cell lines expressing P-gp (Tsuro *et al.*, 1988; Hendricks *et al.*, 1992). However, the relative resistance to the water-soluble camptothecins was modest compared with resistance to doxorubicin, vinblastine and etoposide in the same cell lines.

Two other potentially important mechanisms of resistance have been described. Reduced conversion of the prodrug irinotecan to its active metabolite SN-38 caused loss of efficacy in a cell line selected for resistance to camptothecins (Niimi *et al.*, 1992). Finally, molecular inhibitors of apoptosis such as overexpression of bcl-2 decreased cytotoxicity of camptothecins (Walton *et al.*, 1993). The clinical relevance of all of these mechanisms of resistance remains to be established.

### Discussion

Topoisomerase I inhibitors represent a promising new class of chemotherapeutic agents with a novel mechanism of action. Renewed interest in their study after the initial failure of the parent compound in clinical trials 20 years ago has been driven not only by the understanding of their mechanism of action, but also by an appreciation of structure–activity relationships. The broad anti-tumour activity shown in cell culture and animal studies has been confirmed in clinical phase I/II evaluation of irinotecan and topotecan.

Irinotecan has activity in an array of solid tumours but because of the impressive results in NSCLC and colorectal carcinoma these two tumour types should be the primary focus for the development of combination therapy and randomised trials, at least initially. Preclinical studies have provided helpful information for the development of combination regimens and favour sequential administration of irinotecan with DNA-damaging agents such as cisplatin and topoisomerase II inhibitors such as etoposide. Irinotecan and 5-FU in colorectal carcinoma are also an obvious combination for evaluation and clinical studies are ongoing. In terms of drug delivery, both the weekly and 3-weekly schedules have been shown to be effective; which of the two provides the best therapeutic index is also a question for comparative trials. In addition to the goal of improving efficacy in these and other tumour types, attention must be paid to the toxic effects, especially diarrhoea. Despite maximal therapy diarrhoea remains problematic and will need new solutions before irinotecan can be easily assimilated into routine practice.

Topotecan has a much more favourable toxicity profile than irinotecan but its spectrum of activity in phase II trials is somewhat less impressive. Clearly further studies of topotecan in combination with other effective cytotoxic agents are warranted in SCLC, head and neck cancer, ovarian cancer and possibly breast cancer. Its activity in previously treated ovarian cancer is of particular interest. The results of the recently completed phase III trial comparing the efficacy of topotecan with paclitaxel will be important in determining the enthusiasm for incorporating topotecan into front-line ovarian cancer regimens. In SCLC, the role of topotecan in first-line treatment should be explored and a study addressing this question is currently ongoing in the United States. Phase I/II studies are also underway with topotecan in combination with alkylating agents and topoisomerase II inhibitors similar to those described for irinotecan. At the present time the daily for 5 days schedule appears to offer the best balance of efficacy and toxicity compared with 72 h or 21 day c.i.v. schedules, despite preclinical data favouring prolonged drug exposure. However, clinical studies examining the question of prolonged administration have been limited to tumour sites in which topotecan has not shown impressive activity on the daily × 5 day schedule so these may not have been good models in which to study alternative schedules.

There is no doubt that the clinical data have confirmed that the camptothecins represent an exciting new class of chemotherapeutic agents. The role each analogue will play in improving survival or palliative treatment of specific malignancies is evolving with the present generation of randomised trials but this will take several years to unfold. The data on topotecan and irinotecan have shown how modifications of the parent molecule lead to substantially different efficacy and toxicity profiles. Thus, results of phase II studies with 9-aminocamptothecin and GG 211 will be of great interest.

An additional aspect of the story of camptothecin development deserves comment. It is to point out the critical role that preclinical experiments played in resurrecting the interest in a class of compounds that would otherwise have remained abandoned. The identification of a unique mechanism of action and the chemical studies to determine structure–activity relationships permitted the synthesis of new molecules which were appropriate for clinical evaluation. Furthermore, the clinical trials themselves have been shaped to accommodate new schedules or end points, such as critical blood levels, when preclinical data suggested these factors might play an important role in efficacy. It will be of interest to see if these predictions prove to be accurate as clinical experience matures. Meanwhile the preclinical–clinical dialogue must continue to further our understanding of the determinants of toxicity, resistance and efficacy. Such data will allow optimisation of the use of these agents and permit the development of better analogues in this class.

### References

ABBRUZZESE JL, MADDEN T, SCHMIDT S, EATON G AND RABER MN. (1993). Phase I trial of topotecan (TT) administered by 24-hour infusion without and with G-CSF. (abstract 1957). *Proc. Am. Assoc. Cancer Res.*, **34,** 329.

ABIGERGES D, ARMAND JP, CHABOT CG, DA COSTA L, FADEL E, COTE C, HERAIT P AND GANDIA D. (1994). Irinotecan (CPT-11) high-dose escalation using intensive high-dose loperamide to control diarrhoea. *J. Natl Cancer Inst.*, **86,** 446–449.

ANDOH T, ISHII K, SUZUKI Y, IKEGAMI Y, KUSUNOKI Y, TAKEMOTO Y AND OKADA K. (1987). Characterization of a mammalian mutant with a camptothecin-resistant DNA topoisomerase I. *Proc. Natl Acad. Sci. USA*, **84,** 5565–5569.

ARAKI E, ISHIKAWA M, IIGO M, KOIDE T, ITABASHI M AND HOSHI A. (1993). Relationship between development of diarrhoea and the concentration of SN-38, an active metabolite of CPT-11, in the intestine and the blood plasma of athymic mice following intraperitoneal administration of CPT-11. *Jpn. J. Cancer Res.*, **84,** 697–702.

ARMSTRONG D, ROWINSKY E, DONEHOWER R, ROSENSHEIN N, WALCZAK J AND MCGUIRE W. (1995). A phase II trial of topotecan as salvage therapy in epithelial ovarian cancer. (abstract 769). *Proc. Am. Soc. Clin. Oncol.*, **14,** 275.

BENEDETTI P, FIORANI P, CAPUANI L AND WANG JC. (1993). Camptothecin resistance from a single mutation changing glycine 363 of human DNA topoisomerase I to cysteine. *Cancer Res.*, **53,** 4343–4348.

BERTRAND R, O'CONNOR PM, KERRIGAN D AND POMMIER Y. (1992). Sequential administration of camptothecin and etoposide circumvents the antagonistic cytotoxicity of simultaneous drug administration in slowly growing human colon carcinoma HT-29 cells. *Eur. J. Cancer*, **28A,** 743–748.

BLANEY SM, BALIS FM, COLE DE, CRAIG C, REID JM, AMES MM, KRAILO M, REAMAN G, HAMMOND D AND POPLACK DG. (1993). Pediatric phase I trial and pharmacokinetic study of topotecan administered as a 24-hour continuous infusion. *Cancer Res.*, **53,** 1032–1036.

BONNETERRE J, PION JM, ADENIS M, TUBIANA-HULIN M, TURSZ T, MARTY M AND MATHIEU-BOUE A. (1993). A phase II study of a new camptothecin analogue CPT-11 in previously treated advanced breast cancer patients. (abstract 179). *Proc. Am. Soc. Clin. Oncol.*, **12,** 94.

BUGAT R, SUC E, ROUGIER P, BECOUARN Y, NAIEFF I, YCHOU M, CULINE S, EXTRA JM, ADENIS A, GANEM G, MIOVANNINI M, MERROUCHE M, FERRERO F, CONROY T, DESPAX R, MOUSSEAU I, BEKRADA M, MATHIEU-BOUE A, MAHJOUBI M AND HERAIT P. (1994). CPT-11 (irinotecan) as second line therapy in advanced colorectal cancer (CRC): preliminary results of a multicentric phase II study. (abstract 586). *Proc. Am. Soc. Clin. Oncol.*, **13,** 200.

BUGAT R, ROUGIER P, DOUILLARD JY, BRUNET R, YCHOU M, ADENIS A, MARTY M, SELTZ JF, CONROY T, MAROUCHE Y, GANEM G, NAMER M, BURKI F, MOUSSEAU M AND MAHJOUBI M. (1995). Efficacy of irinotecan HCl (CPT-11) in patients with metastatic colorectal cancer after progression while receiving a 5FU-based chemotherapy. (abstract 567). *Proc. Am. Soc. Clin. Oncol.*, **14,** 222.

BURRIS HA, HANAUSKE AR, JOHNSON RK, MARSHALL MH, KUHN JG, HILSENBECK SG, AND VON HOFF DD. (1992). Activity of topotecan, a new topoisomerase I inhibitor, against human tumor colony-forming units *in vitro*. *J. Natl Cancer Inst.*, **84,** 1816–1820.

BURRIS HA, III, AWADA A, KUHN JG, ECKARDT JR, COBB PW, RINALDI DA, FIELDS SF, SMITH L AND VON HOFF DD. (1994). Phase I and pharmacokinetic studies of topotecan administered as a 72 or 120 h continuous infusion. *Anti-cancer Drugs*, **5,** 394–402.

CHAMPOUX J. (1976). Evidence for an intermediate with single-stand break in the reaction catalyzed by the DNA untwisting enzyme. *Proc. Natl Acad. Sci. USA*, **73,** 3488–3491.

CHANG AY, GARROW G, BOROS L, ASBURY R, PANDYA K AND KENG P. (1995). Clinical and laboratory studies of topotecan in breast cancer. (abstract 118). *Proc. Am. Soc. Clin. Oncol.*, **14,** 105.

CHEN AY, YU C, POTMESIL M, WALL ME, WANI MC AND LIU LF. (1991). Camptothecin overcomes MDR1-mediated resistance in human KB carcinoma cells. *Cancer Res.*, **51,** 6039–6044.

CHEVALLIER B, L'HOMME C, DIERAS V, VENNIN PH, CHAUVERGNE J, REBATTU P, FUMOLEAU P, ROCHE H, KRAKOWSKI Y, LENTZ MA, MATHIEU BOUE A AND VAN GLABBEKE M. (1995). Phase II trial of CPT11 in advanced cervical carcinoma. (abstract 737). *Proc. Am. Clin. Oncol.*, **14,** 267.

CLAVEL M, MATHIEU-BOUE A, DUMORTIER A, CHABOT GG, COTE C, BISSERY MC AND MARTY M. (1992). Phase I study of CPT-11 administered as a daily infusion for 3 consecutive days. (abstract 1568). *Proc. Am. Assoc. Cancer Res.*, **33,** 262.

CONTI JA, KAMENY N, SALTZ L, TONG W, CHOU TC AND PULLIAM M. (1994). Irinotecan (CPT-11) is an active agent in untreated patients (pts) with metastatic colorectal cancer (CRC). (abstract 565). *Proc. Am. Soc. Clin. Oncol.*, **13,** 195.

CREEMERS GJ, WANDERS J, CALABRESI F, VALENTIN S, DIRIX LY, SCHOFFSK IP, FRANKLIN H, MCDONALD M AND VERWEIJ J. (1994). Topotecan in colorectal cancer, a phase II study of the EORTC early clinical trials group. (suppl. 5, abstract 464). *Ann. Oncol.*, **5,** 191.

CREEMERS GJ, SCHELLENS JHM, PLANTING ASTH, VD BURG MEL, DEBOER-DENNERT M, HARTEVELD M, MCDONALD M, STOTER G AND VERWEIJ J. (1995). Cumulative myelosuppression of topotecan (T) administered as a 21-day continuous infusion in patients with colorectal cancer. (abstract 354). *Proc. Am. Soc. Clin. Oncol.*, **14,** 167.

DAHUT W, BRILLHART N, TAKIMOTO C, ALLEGRA C, HAMILTON JM, SORENSEN S, ARBUCK S, CHEN A AND GREM J. (1994). A phase I trial of 9-aminocamptothecin (9-AC) in adult patients with solid tumors. (abstract 345). *Proc. Am. Soc. Clin. Oncol.*, **13,** 138.

DE FORNI M, BUGAT R, CHABOT GG, CULINE S, EXTRA J-M, GOUYETTE A, MADELAINE I, MARTY ME AND MATHIEU-BOUE A. (1994). Phase I and pharmacokinetic study of the camptothecin derivative irinotecan administered on a weekly schedule in cancer patients. *Cancer Res.*, **54,** 4347–4354.

DEL BINO G, LASSOTA P AND DARZYNKIEWICZ Z. (1991). The S-phase cytotoxicity of camptothecin. *Exp. Cell Res.*, **193,** 27–35.

DEL BINO G, BRUNO S, YI PN AND DARZYNKIEWICZ Z. (1992). Apoptotic cell death triggered by camptothecin or teniposide. The cell cycle specificity and effects of ionizing radiation. *Cell Proliferation*, **25,** 537–548.

DOUILLARD JY, IBRAHIM N, RIVIERE A, SPAETH D, CHOMY P, SOUSSAN K AND MATHIEU-BOUE A. (1995). Phase II study of CPT-11 in non small cell lung cancer (NSCLC). (abstract 1118). *Proc. Am. Soc. Clin. Oncol.*, **14,** 365.

ECKARDT JR, BURRIS HA, VON HOFF DD, RODRIGUEZ GI, FIELDS SM, ROTHENBERG ML, MOORE TD, HODGES S, WEISS GR, COBB P, RINALDI D, KUHN JG, FORD J AND GANAPATHI R. (1994). Measurement of tumor topoisomerase I and II levels during the sequential administration of topotecan and etoposide. (abstract 358). *Proc. Am. Soc. Clin. Oncol.*, **13**, 141.

ECKARDT JR, RODRIGUEZ GI, BURRIS HA, WISSEL PS, FIELDS SM, ROTHENBERG ML, SMITH L, THURMAN A, KUNKA RL, DEPEE SP, LITTLEFIELD D, WHITE LJ AND VON HOFF DD. (1995). A phase I and pharmacokinetic study of the topoisomerase I inhibitor GG211. (abstract 1544). *Proc. Am. Soc. Clin. Oncol.*, **14**, 476.

EISENHAUER EA, WAINMAN N, BOOS G, MACDONALD D AND BRAMWELL V. (1994). Phase II trials of topotecan in patients (pts) with malignant glioma and soft tissue sarcoma. (abstract 488). *Proc. Am. Soc. Clin. Oncol.*, **13**, 175.

EMERSON DL, MCINTYRE G, LUZZIO MJ AND WISSEL PS. (1994). Pre-clinical anti tumor activity of a novel water-soluble camptothecin analog, (GI147211C). (suppl. 5, abstract 441). *Ann. Oncol.*, **5**, 185.

EMERSON DL, BESTERMAN JM, BROWN HR, EVANS MG, LEITNER PP, LUZZIO MJ, SHAFFER JE, STERNBACH DD, UEHLING D AND VUONG A. (1995). *In vivo* antitumor activity of two new seven-substituted water-soluble camptothecin analogues. *Cancer Res.*, **55**, 603–609.

FRIEDMAN HS, HOUGHTON PJ, SCHOLD SC, KEIR S AND BIGNER DD. (1994). Activity of 9-dimethylaminomethyl-10-hydroxy-camptothecin against pediatric and adult central nervous system tumor xenografts. *Cancer Chemother. Pharmacol.*, **34**, 171–174.

FUJIWARA Y, YAMAKIDO M, FUKUOKA M, KUDOH S, FURUSE K, IKEGAMI H, ARIYOSHE Y FOR THE WEST JAPAN LUNG CANCER STUDY GROUP. (1994). Phase II study of irinotecan (CPT-11) and cisplatin (CDDP) in patients with small cell lung cancer (SCLC). (abstract 1110). *Proc. Am. Soc. Clin. Oncol.*, **13**, 335.

FUKUOKA M, NEGORO S, NIITANI H, FURUE H, HASEGAWA K, HARA Y, HARA N. AND TAGUCHI T. (1990). A phase I study of weekly administration of CPT-11 in lung cancer. *Gan to Kagaku Ryoho*, **17**, 993–997.

FUKUOKA M., NIITANI H, SUZUKI A, MOTOMIYA M, HASEGAWA K, NISHIWAKI Y, KURIYAMA T, ARIYOSHI Y, NEGORO S, MASUDA N, NAKAJIMA S, TAGUCHI T. (1992). A phase II study of CPT-11, a new derivative of camptothecin, for previously untreated non-small-cell lung cancer. *J. Clin. Oncol.*, **10**, 16–20.

FURUTA T AND YOKOKURA T. (1990). Effect of administration schedules on the antitumor activity of CPT-11, a camptothecin derivative. *Gan to Kagaku Ryoho*, **17**, 121–130.

FUTATSUKI K, WAKUI A, NAKAO I, SAKATA Y, KAMBE M, SHIMADA Y, YOSHINO M, TAGUCHI T AND OGAWA N. (1994). Late phase II study of irinotecan hydrochloride (CPT-11) in advanced gastric cancer. CPT-11 Gastrointestinal Cancer Study Group. *Gan to Kagaku Ryoho*, **21**, 1033–1038.

GANDIA D, ABIGERGES D, ARMAND JP, CHABOT G, DA COSTA L, DE FORNI M, MATHIEU-BOUE A AND HERAIT P. (1993). CPT-11-induced cholinergic effects in cancer patients. *J. Clin. Oncol.*, **11**, 196–197.

GIANTONIO BJ, KOSIEROWSKI R, RAMSEY HE, FOX SC, MCALEER CA, ROETHKE S, OZOLS RF AND HUDES GR. (1993). Phase II study of topotecan (TT) for hormone refractory prostate cancer (HRPC). (abstract 774). *Proc. Am. Soc. Clin. Oncol.*, **12**, 247.

GIOVANELLA BC, STEHLIN JS, WALL ME, WANI MC, NICHOLAS AW, LIU LF, SILBER R AND POTMESIL M. (1989). DNA topoisomerase I-targeted chemotherapy of human colon cancer in xenografts. *Science*, **246**, 1046–1048.

GIOVANELLA BC, HINZ HR, KOZIELSKI AJ, STEHLIN JS JR, SILBER R AND POTMESIL M. (1991). Complete growth inhibition of human cancer xenografts in nude mice by treatment with 20-(s)-camptothecin. *Cancer Res.*, **51**, 3052–3055.

GONG J, LI X AND DARZYNKIEWICZ Z. (1993). Different patterns of apoptosis of HL-60 cells induced by cycloheximide and camptothecin. *J. Cell Physiol.*, **157**, 263–270.

GOTO K, NISHIWAKI Y, SAIJO N, NAKABAYASHI T, KAWAKAMI Y, FUJITA A, TOBISE K, ABE S, SUZUKI S, TSUCHIZY S, TAKAHASHI Y, HAYASHI I, NODA K, KURITA Y, MATSUDA Y, TAMURA T AND SHIMOYAMA M. (1995). A phase II study of irinotecan (CPT-11) and etoposide (VP-16) for metastatic non-small cell lung cancer (NSCLC): Japanese Clinical Oncology Group (JCOG) trial. (abstract 1108). *Proc. Am. Soc. Clin. Oncol.*, **14**, 362.

GOTTLEIB JA AND LUCE JK. (1972). Treatment of malignant melanoma with camptothecin (MSC-100880). *Cancer Chemother. Rep.*, **56**, 103–105.

GROCHOW LB, SLICHENMYER W, ROWINSKY E, DONEHOWER R, FORASTIERE A AND CHEN T-L. (1994). Phase I clinical and pharmacologic study of topotecan (top) in patients with hepatic or renal dysfunction. (suppl. 5, abstract 462). *Ann. Oncol.*, **5**, 191.

GUPTA E, LESTINGI TM, MICK R, RAMIREZ J, VOKES EE AND RATAIN MJ. (1994). Metabolic fate of irinotecan in humans: correlation of glucuronidation with diarrhea. *Cancer Res.*, **54**, 3723–3725.

HAAS NB, LACRETA FP, WALCZAK J, HUDES GR, BRENNAN JM, OZOLS RF AND O'DWYER PJ. (1994). Phase I/pharmacokinetic study of topotecan by 24-hour continuous infusion weekly. *Cancer Res.*, **54**, 1220–1226.

HAGIPANTELLI R, SALIBA F, MISSET JL, GIACCHETTI S, BRAIN E, BERTHEAULT-CVITKOVIC F, VASSAL G, BONNAY M, BASTIAN G, COTE C, MAHJOUBI M, HERAIT P AND CVITKOVIC E. (1995). Pathophysiology and therapy of irinotecan (CPT-11) induced delayed onset diarrhea (DD): a prospective assessment. (abstract 1499). *Proc. Am. Soc. Clin. Oncol.*, **14**, 464.

HASEGAWA K, HISHIMURA R, FUKUOKA M, FURUSE K, HASEGAWA K, HINO M AND NIITANI H. (1993). Phase I and pharmacologic evaluation of topotecan on a 30 minute infusion. (abstract 2514). *Proc. Am. Assoc. Cancer Res.*, **34**, 421.

HENDRICKS CB, ROWINSKY EK, GROCHOW LB, DONEHOWER RC AND KAUFMANN SH. (1992). Effect of P-glycoprotein expression on the accumulation and cytotoxicity of topotecan (SK&F 104864), a new camptothecin analogue. *Cancer Res.*, **52**, 2268–2278.

HERTZBERG RP, CARANFA MJ AND HECHT SM. (1989*a*). On the mechanism of topoisomerase I inhibition by camptothecin: evidence for binding to an enzyme-DNA complex. *Biochemistry*, **28**, 4629–4638.

HERTZBERG RP, CARANFA MJ, HOLDEN KG, JAKAS DR, GALLAGHER G, MATTERN MR, MONG SM, BARTUS JO, JOHNSON RK AND KINGSBURY WD. (1989*b*). Modification of the hydroxy lactone ring of camptothecin: inhibition of mammalian topoisomerase I and biological activity. *J. Med. Chem.*, **32**, 715–720.

HOCHSTER H, LIEBES L, SPEYER J, SORICH J, TAUBES B, ORATZ R, WERNZ J, CHACHOUA A, RAPHAEL B, VINCI RZ AND BLOOM RH. (1994). Phase I trial of low-dose continuous topotecan infusion in patients with cancer: an active and well-tolerated regimen. *J. Clin. Oncol.*, **12**, 553–559.

HOUGHTON PJ, CHESHIRE PJ, HALLMAN JC, BISSERY MC, MATHIEU-BOUE A AND HOUGHTON JA. (1993). Therapeutic efficacy of the topoisomerase I inhibitor 7-ethyl-10-(4-[1-piperidino]-1-piperidino)-carbonyloxy-camptothecin against human tumor xenografts: lack of cross-resistance *in vivo* in tumours with acquired resistance to the topoisomerase I inhibitor 9-dimethylaminomethyl-10-hydroxycamptothecin. *Cancer Res.*, **53**, 2823–2829.

HSIANG YH AND LIU LF. (1988). Identification of mammalian DNA topoisomerase I as an intracellular target of the anticancer drug camptothecin. *Cancer Res.*, **48**, 1722–1726.

HSIANG YH, LIU LF, WALL ME, WANI MC, NICHOLAS AW, MANIKUMAR G, KIRSCHENBAUM S, SILBER R AND POTMESIL M. (1989). DNA topoisomerase I-mediated DNA cleavage and cytotoxicity of camptothecin analogues. *Cancer Res.*, **49**, 4385–4389.

HUTSON PR, KIM K, JOHNSON D AND SCHILLER JH. (1995). Pharmacodynamic evaluation of the response of extensive stage small cell lung cancer to topotecan. (abstract 1481). *Proc. Am. Soc. Clin. Oncol.*, **14**, 460.

ILSON D, MOTZER RJ, O'MOORE P, NANUS D, BOSL GJ (1993). A phase II trial of topotecan in advanced renal cell carcinoma. (abstract 779). *Proc. Am. Soc. Clin. Oncol.*, **12**, 248.

JANIK J, MILLER L, SMITH J, II, KOPP W, ALVORD G, GAUSE B, CURIT B, URBA WJ AND LONGO DL. (1993). Prechemotherapy granulocyte-macrophage colony stimulating factor (GM-CSF) prevents topotecan-induced neutropenia. (abstract 1507). *Proc. Am. Soc. Clin. Oncol.*, **12**, 437.

JAXEL C, KOHN KW, WANI MC, WALL ME AND POMMIER Y. (1989). Structure–activity study of the actions of camptothecin derivatives on mammalian topoisomerase I: evidence for a specific receptor site and a relation to antitumor activity. *Cancer Res.*, **49**, 1465–1469.

KANG MC, BANKSTON DD, ERICKSON GA, FANG FG, LACKEY JW, LEWIS GS, LICHTY ME, LOWERY MW, MADER C, MCDOUGAL DL, MOOK RA, PARTRIDGE JJ AND XIE S. (1993). Total synthesis of the novel water soluble camptothecin analog GI 147211C, a potent topoisomerase inhibitor. (abstract 1976). *Proc. Am. Assoc. Cancer Res.*, **34**, 332.

KANO Y, SUZUKI K, AKUTSU M, SUDA K, INOUE Y, YOSHIDA M, SAKAMOTO S AND MIURA Y. (1992). Effects of CPT-11 in combination with other anti-cancer agents in culture. *Int. J. Cancer*, **50**, 604–610.

KANTARJIAN HM, BERAN M, ELLIS A, ZWELLING L, O'BRIEN S, CAZENAVE L, KOLLER C, RIOS MB, PLUNKETT W, KEATING MJ AND ESTEY EH. (1993). Phase I study of Topotecan, a new topoisomerase I inhibitor, in patients with refractory or relapsed acute leukemia. *Blood*, **81**, 1146–1151.

KAUFMANN SH. (1991). Antagonism between camptothecin and topoisomerase II-directed chemotherapeutic agents in a human leukemia cell line. *Cancer Res.*, **51**, 1129–1136.

KAVANAGH JJ, KUDELKA AP, EDWARDS CE, GIRTANNER RE, DURIVAGE HF AND HOWELL ET. (1994). CPT-II (irinotecan): Phase II study in refractory squamous cell carcinoma of the cervix. *Proc. Am. Assoc. Cancer Res.*, **35**, 234.

KAWATO Y, AONUMA M, HIROTA Y, KUGA H AND SATO K. (1991*a*). Intracellular roles of SN-38, a metabolite of the camptothecin derivative CPT-11, in the antitumor effect of CPT-11. *Cancer Res.*, **51**, 4187–4191.

KAWATO Y, FURUTA T, AONUMA M, YASUOKA M, YOKOKURA T AND MATSUMOTO K. (1991*b*). Antitumor activity of a camptothecin derivative, CPT-11, against human tumor xenografts in nude mice. *Cancer Chemother. Pharmacol.*, **28**, 192–198.

KAWATO Y, SEKIGUCHI M, AKAHANE K, TSUTOMI Y, HIROTA Y, KUGA H, SUZUKI W, HAKUSUI H AND SATO K. (1993). Inhibitory activity of camptothecin derivatives against acetylcholinesterase in dogs and their binding activity to acetylcholine receptors in rats. *J. Pharm. Pharmacol.*, **45**, 444–448.

KIM JH, KIM SH, KOLOZSVARY A AND KHIL MS. (1992). Potentiation of radiation response in human carcinoma cells *in vitro* and murine fibrosarcoma *in vivo* by topotecan, an inhibitor of DNA topoisomerase I. *Int. J. Rad. Oncol. Biol. Phys.*, **22**, 515–518.

KIM R, HIRABAYSHI N, NISHIYAMA M, JINUSHI K, TOGE T AND OKADA K. (1992). Experimental studies on biochemical modulation targeting topoisomerase I and II in human tumour xenografts in nude mice. *Int. J. Cancer*, **50**, 760–766.

KUDELKA A, EDWARDS C, FREEDMAN R, WALLIN B, HORD M, HOWELL E, HARPER K, RABER M AND KAVANAGH J. (1993). An open phase II study to evaluate the efficacy and toxicity of topotecan administered intravenously as 5 daily infusions every 21 days to women with advanced epithelial ovarian carcinoma. (abstract 821). *Proc. Am. Soc. Clin. Oncol.*, **12**, 259.

KUNIMOTO T, NITTA K, TANAKA T, UEHARA N, BABA H, TAKEUCHI M, YOKOKURA T, SAWADA S, MIYASAKA T AND MUTAI M. (1987). Antitumor activity of 7-ethyl-10-[4-(1-piperidino)-1-piperidino]carbonyloxy-camptothecin, a novel water-soluble derivative of camptothecin, against murine tumors. *Cancer Res.*, **47**, 5944–5947.

KURAISHI Y, SANO M, HIRANO A, DOBASHI N, MIZUNUMA N, FUNAKOSHI T, TAKASAKI N, KOBAUASHI T, ISOGAI Y, YAMASHITA T, AIBA K AND OGAWA M. (1992). *In vitro* combination effect of CPT-11, a new camptothecin derivative in human non-small cell lung cancer cell lines. (abstract 308). *Proc. Am. Soc. Clin. Oncol.*, **11**, 122.

LAW TM, ILSON DH AND MOTZER RJ. (1994). Phase II trial of topotecan in patients with advanced renal cell carcinoma. *Invest. New Drugs*, **12**, 143–145.

LESTINGI TM, VOKES EE, GRAY W, SCHILSKY RL, VOGELZANG NJ, MICK R, GUPTA E AND RATAIN MJ. (1995). A phase I trial of CPT-11 in solid tumors with G-CSF and antidiarrheal support. (abstract 1536). *Proc. Am. Soc. Clin. Oncol.*, **14**, 480.

LYNCH TJJR, KALISH L, STRAUSS G, ELIAS A, SKARIN A, SHULMAN LN, POSNER M AND FREI E. (1994). Phase II study of topotecan in metastatic non-small-cell lung cancer. *J. Clin. Oncol.*, **12**, 347–352.

MCCABE FL AND JOHNSON RK. (1994). Comparative activity of oral and parenteral topotecan in murine tumor models: efficacy of oral topotecan. *Cancer Invest.*, **12**, 308–313.

MADDEN KR AND CHAMPOUX JJ. (1992). Overexpression of human topoisomerase I in baby hamster kidney cells: hypersensitivity of clonal isolates to camptothecin. *Cancer Res.*, **52**, 525–532.

MAKSYMIUK AW, JUNG S-H, MARSCHKE RF, JR, NAIR S AND JETT JR. (1995). Phase II trial of topotecan in pleural mesothelioma: a North Central Cancer Treatment Group (NCCTG) trial. (abstract 1383). *Proc. Am. Soc. Clin. Oncol.*, **14**, 435.

MASUDA N, FUKUOKA M, KUSUNOKI Y, MATSUI K, TAKIFUJI N, KUDOH S, NEGORO S, NISHIOKA M, NAKAGAWA K AND TAKADA M. (1992). CPT-11: a new derivative of camptothecin for the treatment of refractory or relapsed small-cell lung cancer. *J. Clin. Oncol.*, **10**, 1225–1229.

MATTERN MR, HOFMANN GA, MCCABE FL AND JOHNSON RK. (1991). Synergistic cell killing by ionizing radiation and topoisomerase I inhibitor topotecan (SK&F 104864). *Cancer Res.*, **51**, 5813–5816.

MINAMI H, BEIJNAN JH, VERWEIJ J, RATAIN MJ. (1996). Limited sampling model for area under the concentration time curve of total topotecan. *Clin Cancer Res.*, **2**, 43–46.

MOERTEL CG, SCHUTT AJ, REITEMEIER RJ AND HAHN RG. (1972). Phase II study of camptothecin (NSC-100880) in the treatment of advanced gastrointestinal cancer. *Cancer Chemother. Rep.*, **56**, 95–101.

MUGGIA FM, CREAVEN PJ, HANSEN HH, COHEN MH AND SELAWRY OS. (1972). Phase I clinical trial of weekly and daily treatment with camptothecin (NSC-100880): correlation with preclinical studies. *Cancer Chemother. Rep.*, **56**, 515–521.

MURPHY B, SALTZ L, SIROTT M, YOUNG C, TONG W, TROCHANOWSKI B, TOOMASI R AND KELSEN D. (1992). Granulocyte-colony stimulating factor (G-CSF) does not increase the maximum tolerated dose (MTD) in a phase I study of topotecan. (abstract 379). *Proc. Am. Soc. Clin. Oncol.*, **11**, 139.

NARITA M, NAGAI E, HAGIWARA H, ABURADA M, YOKOI T AND KAMATAKI T. (1993). Inhibition of beta-glucuronidase by natural glucuronides of kampo medicines using glucuronide of SN-38 (7-ethyl-10-hydroxycamptothecin) as a substrate. *Xenobiotica*, **23**, 5–10.

NEGORO S, FUKUOKA M, MASUDA N, TAKADA M, KUSUNOKI Y, MATSUI K, TAKIFUJI N, KUDOH S, NIITANI H AND TAGUCHI T. (1991*a*). Phase I study of weekly intravenous infusions of CPT-11, a new derivative of camptothecin, in the treatment of advanced non-small-cell lung cancer. *J. Nat. Cancer Inst.*, **83**, 1164–1168.

NEGORO S, FUKUOKA M, NIITANI H, SUZUKI A, NAKABAYASHI T, KIMURA M, MOTOMIYA M, KURITA Y, HASEGAWA K, KURIYAMA T, NISHIWAKI Y, OGAWA M, NAKAO I, SAIJO N, OBA K, FURUE H, ARIYOSHI Y, SHIMOKATA K, FURUSE K, NAKAJIMA S, IRIE K, KIMURA I, OGURA T, FUJII M, HARA N, HARA Y, NAKAO S, ARAKI J, MIYATA Y, TAGUCHI T. (1991*b*). A phase II study of CPT-11, a camptothecin derivative, in patients with primary lung cancer. CPT-11 Cooperative Study Group. *Gan to Kagaku Ryoho*, **18**, 1013–1019.

NIIMI S, NAKAGAWA K, SUGIMOTO Y, NISHIO K, FUJIWARA Y, YOKOYAMA S, TERASHIMA Y AND SAIJO N. (1992). Mechanism of cross-resistance to a camptothecin analogue (CPT-11) in a human ovarian cancer cell line selected by cisplatin. *Cancer Res.*, **52**, 328–333.

O'CONNOR PM, NIEVES-NEIRA W, KERRIGAN D, BERTRAND R, GOLDMAN J, KOHN KW AND POMMIER Y. (1991). S-phase population analysis does not correlate with the cytotoxicity of camptothecin and 10,11-methylenedioxycamptothecin in human colon carcinoma HT-29 cells. *Cancer Commun.*, **3**, 233–240.

O'DWYER P, CASSIDY J, KUNKA R, PAS-AREZ L, KAYE S, DEPEE S, LITTLEFIELD D, DEMARIA D, SELINGER K, BERANEK P, COLLIS P AND WISSEL P. (1995). Phase I trial of GG211, a new topoisomerase inhibitor, using a 72 hour continuous infusion (CI). (abstract 1525). *Proc. Am. Soc. Clin. Oncol.*, **14**, 471.

OHE Y, SASAKI Y, SHINKAI T, EGUCHI K, TAMURA T, KOJIMA A, KUNIKANE H, OKAMOTO H, KARATO A, OHMATSU H, KANAZAWA F AND NAGAHIRO S. (1992). Phase I study and pharmacokinetics of CPT-11 with 5-day continuous infusion. *J. Natl Cancer Inst.*, **84**, 972–974.

OHNO R, OKADA K, MASAOKA T, KURAMOTO A, ARIMA T, YOSHIDA Y, ARIYOSHI H, ICHIMARU M, SAKAI Y, OGURO M, ITO Y, MORISHIMA Y, YOKOMAKU S AND OTA K. (1990). An early phase II study of CPT-11: a new derivative of camptothecin, for the treatment of leukemia and lymphoma. *J. Clin. Oncol.*, **8**, 1907–1912.

OTA K, OHNO R, SHIRAKAWA S, MASAOKA T, OKADA K, OHASHI Y AND TAGUCHI T. (1994). Late phase II clinical study of irinotecan hydrochloride (CPT-11) in the treatment of malignant lymphoma and acute leukemia. The CPT-11 Research Group for Hematological Malignancies. *Gan to Kagaku Ryoho*, **21**, 1047–1055.

PANTAZIS P, HINZ HR, MENDOZA JT, KOZIELSKI AJ, WILLIAMS LJ, JR, STEHLIN JS, JR, AND GIOVANELLA BC. (1992). Complete inhibition of growth followed by death of human malignant melanoma cells *in vitro* and regression of human melanoma xenografts in immunodeficient mice induced by camptothecins. *Cancer Res.*, **52**, 3980–3987.

PEREZ-SOLER R, GLISSON BS, KANE J, LEE J, RABER MN AND HONG WK. (1994). Phase II study of topotecan in patients with non-small cell lung cancer (NSCLC) previously untreated. (abstract 1223). *Proc. Am. Soc. Clin. Oncol.*, **13**, 363.

PEREZ-SOLER R, GLISSON BS, LEE JS, FOSSELLA FV, LIPPMAN SM, HUBER MH, SHIN DM, MURPHY WK AND HONG WK. (1995). Phase II study of topotecan in patients with small cell lung cancer (SCLS) refractory to etoposide. (abstract 1078). *Proc. Am. Soc. Clin. Oncol.*, **14**, 355.

PITOT HC, WENDER D, O'CONNELL NJ, WIEAND HS AND MAILLIARD JA. (1994). A phase II trial of CPT-11 (irinotecan) in patients with metastatic colorectal carcinoma: a North Central Cancer Treatment Group (NCCTG) study. (abstract 573). *Proc. Am. Soc. Clin. Oncol.*, **13**, 197.

PLAXE S, CHRISTEN R, O'QUIGLEY J, BRALY P, FREDDO J, McCLAY E, HEATH D AND HOWELL S. (1993). Phase I trial of intraperitoneal topotecan. (abstract 360). *Proc. Am. Soc. Clin. Oncol.*, **12**, 140.

POMMIER Y. (1993). DNA topoisomerase I and II in cancer chemotherapy: update and perspectives. *Cancer Chemother. Pharmacol.*, **32**, 103–108.

POTKUL RK, PRICE FV, BAILEY H, GELDER M, ROSENBLUTH R AND DURIVAGE HJ. (1995). Irinotecan (CPT-11) in advanced squamous cell carcinoma of the cervix (phase II). (abstract 785). *Proc. Am. Soc. Clin. Oncol.*, **14**, 279.

POTMESIL M. (1994). Camptothecins: from bench research to hospital wards. *Cancer Res.*, **54**, 1431–1439.

PRATT CB, STEWART C, SANTANA VM, BOWMAN L, FURMAN W, OCHS J, MARINA N, KUTTESCH JF, HEIDEMAN R, SANDLUND JT, AVERY L AND MEYER WH. (1994). Phase I study of topotecan for pediatric patients with malignant solid tumors. *J. Clin. Oncol.*, **12**, 539–543.

ROBERT F, WHEELER RH, MOLTHROP DC, GREENE P AND CHEN S. (1994). Phase II study of topotecan in advanced head and neck cancer: identification of an active new agent. (abstract 905). *Proc. Am. Soc. Clin. Oncol.*, **13**, 281.

ROTHENBERG ML, KUHN JG, BURRIS HA, NELSON J, ECKARDT JR, TRISTAN-MORALES M, HILSENBECK SG, WEISS GR, SMITH LS, RODRIGUEZ GI, ROCK MK AND VON HOFF DD. (1993). Phase I and pharmacokinetic trial of weekly CPT-11. *J. Clin. Oncol.*, **11**, 2194–2204.

ROTHENBERG ML, ECKARDT JR, BURRIS HA, III, NELSON J, KUHN JG, CHEN SF, HILSENBECK SB, CLARK GM, FIELDS SM, RODRIGUEZ GI, WEISS GR, SMITH LS, THURMAN AM, ECKHARDT SG, RINALDI DA, PEREZ E AND VON HOFF DD. (1994). Irinotecan (CPT-11) as second-line therapy for patients with 5-FU refractory colorectal cancer. (abstract 578). *Proc. Am. Soc. Clin. Oncol.*, **13**, 198.

ROUGIER PH, CULINE S, BUGAT R, BRUNET P, DOUILLARD JY, YCHOU M, MARTY M, BONNETERRE J, GANEM G, SEITZ JF, NEGRIER S, NAMER M, CONROY T, BURKI F, SHEARRER A, DROZ JP, MATHIEU-BOUE A, MAHJOUBI M AND HERAIT P. (1994). Multicentric phase II study of first line CPT-11 (irinotecan) in advanced colorectal cancer (CRC). (abstract 585). *Proc. Am. Soc. Clin. Oncol.*, **13**, 200.

ROWINSKY EK, GROCHOW LB, HENDRICKS CB, ETTINGER DS, FORASTIERE AA, HUROWITZ LA, McGUIRE WP, SARTORIUS SE, LUBEJKO BG, KAUFMANN SH AND DONEHOWER RC. (1992). Phase I and pharmacologic study of topotecan: a novel topoisomerase I inhibitor. *J. Clin. Oncol.*, **10**, 647–656.

ROWINSKY EK, GROCHOW LB, ETTINGER DS, SARTORIUS SE, LUBEJKO BG, CHEN TL, ROCK MK AND DONEHOWER RC. (1994*a*). Phase I and pharmacological study of the novel topoisomerase I inhibitor 7-ethyl-10-[4-(1-piperidino)-1-piperidino]carbonyloxycamptothecin (CPT-11) administered as a ninety-minute infusion every 3 weeks. *Cancer Res.*, **54**, 427–436.

ROWINSKY E, GROCHOW L, KAUFMANN S, BOWLING K, BUBEJKO B, CHEN T, ETTINGER D, PEEREBOOM D, SARTORIUS S AND DONEHOWER R. (1994*b*). Sequence-dependent effects of topotecan (T) and cisplatin (C) in a phase I and pharmacology study. (abstract 468). *Ann. Oncol.*, **5**, 192.

RUBIN E, WOOD V, BHARTI A, TRITES D, LYNCH C AND KUFE D. (1994). A phase I trial of 9-amino-camptothecin (9-AC). (abstract 1465). *Proc. Am. Assoc. Cancer Res.*, **35**, 245.

SABIERS JH, BERGER NA, BERGER SJ, HAAGA JR, HOPPEL LL AND WILLSON JKV. (1993). Phase I trial of topotecan administered as a 72 hour infusion. (abstract 2541). *Proc. Am. Assoc. Cancer Res.*, **34**, 426.

SAKATA Y, SHIMADA Y, YOSHINO M, KAMBE M, FUTATSUKI K, NAKAO I, OGAWA N, WAKUI A AND TAGUCHI T. (1994). A late phase II study of CPT-11, irinotecan hydrochloride, in patients with advanced pancreatic cancer. CPT-11 Study Group on Gastrointestinal Cancer. *Gan to Kagaku Ryoho*, **21**, 1039–1046.

SALTZ L, SIROTT M, YOUNG C, TONG W, NIEDZWIECKI D, TZY-JYUN Y, TAO Y, TROCHANOWSKI B, WRIGHT P, BARBOSA K, TOOMASI F AND KELSEN D. (1993). Phase I clinical and pharmacology study of topotecan given daily for 5 consecutive days to patients with advanced solid tumours, with attempt at dose intensification using recombinant granulocyte colony-stimulating factor. *J. Natl Cancer Inst.*, **85**, 1499–1507.

SCHER R, LUSCH CJ, GREEN F, KOSIEROWSKI R, SIMMONDS M, ENGSTROM PF AND O'DWYER PJ. (1994). Phase II trial of topotecan in advanced pancreatic cancer. (suppl.5, abstract 463). *Ann. Oncol.*, **5**, 191.

SCHILLER JH, KIM K, JOHNSON D AND THE EASTERN COOPERATIVE ONCOLOGY GROUP. (1994). Phase II study of topotecan in extensive stage small cell lung cancer. (abstract 1093). *Proc. Am. Soc. Clin. Oncol.*, **13**, 330.

SHIMADA Y, YOSHINO M, WAKUI A, NAKAO I, FUTATSUKI K, SAKATA Y, KAMBE M, TAGUCHI T AND OGAWA N. (1993). Phase II study of CPT-11, a new camptothecin derivative, in metastatic colorectal cancer. CPT-11 Gastrointestinal Cancer Study Group. *J. Clin. Oncol.*, **11**, 909–913.

SLICHENMYER WJ, ROWINSKY EK, DONEHOWER RC AND KAUFMANN SH. (1993). The current status of camptothecin analogues as antitumor agents. *J. Natl Cancer Inst.*, **85**, 271–291.

SUGARMAN SM, AJANI JA, DAUGHERTY K, WINN R, LANZOTTI V, BEARDEN JD AND ABBRUZZESE LL. (1994*a*). A phase II trial of topotecan (TPT) for the treatment of advanced, measurable colorectal cancer. (abstract 686). *Proc. Am. Soc. Clin. Oncol.*, **13**, 225.

SUGARMAN SM, PAZDUR R, DAUGHERTY K, EVANS D, WINN R, DUBOVSKY D, GOODWIN JW AND ABBRUZZESE JL. (1994*b*). A phase II trial of topotecan (TPT) for the treatment of unresectable pancreatic cancer (PC). (abstract 684). *Proc. Am. Soc. Clin. Oncol.*, **13**, 224.

SUGIMOTO Y, TSUKAHARA S, OH-HARA T, LIU LF AND TSURUO T. (1990*a*). Elevated expression of DNA topoisomerase II in camptothecin-resistant human tumor cell lines. *Cancer Res.*, **50**, 7962–7965.

SUGIMOTO Y, TSUKAHARA S, OH-HARA T, ISOE T AND TSURUO T. (1990*b*). Decreased expression of DNA topoisomerase I in camptothecin-resistant tumor cell lines as determined by a monoclonal antibody. *Cancer Res.*, **50**, 6925–6930.

SUPKO JG AND MALSPEIS L. (1993). Pharmacokinetics of the 9-amino and 10,11-methylenedioxy derivatives of camptothecin in mice. *Cancer Res.*, **53**, 3062–3069.

TAGUCHI T, WAKUI A, HASEGAWA K, NIITANI H, FURUE H, OHTA K AND HATTORI T. (1990). Phase I clinical study of CPT-11. Research group of CPT-11. *Gan to Kagaku Ryoho*, **17**, 115–120.

TAGUCHI T, TOMINAGA T, OGAWA M, ISHIDA T, MORIMOTO K AND OGAWA N. (1994). A late phase II study of CPT-11 (irinotecan) in advanced breast cancer. CPT-11 Study Group on Breast Cancer. *Gan to Kagaku Ryoho*, **21**, 1017–1024.

TAKEUCHI S, DOBASHI K, FUKIMOTO S, TANAKA K, SUZUKI M, TERASHIMA Y, HASUMI K, AKIYA K, NEGISHI Y, TAMAYA T AND ET AL. (1991). A late phase II study of CPT-11 on uterine cervical cancer and ovarian cancer. Research Groups of CPT-11 in Gynecologic Cancers. *Gan to Kagaku Ryoho*, **18**, 1681–1689.

TAKIMOTO CH, KLECKER RW, DAHUT WL, BRILLHART N, YEE LK, STRON JM, NAKASHIMA H, LIEBERMAN R, ALLEGRA CJ AND GREM JL. (1994). Preliminary pharmacokinetics of the active lactone form of 9-aminocamptothecin using a sensitive new HPLC assay. (abstract 1443). *Proc. Am. Assoc. Cancer Res.*, **35**, 242.

TAN KB, MATTERN MR, ENG WK, McCABE FL AND JOHNSON RK. (1989). Nonproductive rearrangement of DNA topoisomerase I and II genes: correlation with resistance to topoisomerase inhibitors. *J. Natl Cancer Inst.*, **81**, 1732–1735.

TANIZAWA A, BEITRAND R, KOHLHAGEN G, TABUCHI A, JENKINS J AND POMMIER Y. (1993). Cloning of Chinese hamster DNA topoisomerase I cDNA and identification of a single point mutation responsible for camptothecin resistance. *J. Biol. Chem.*, **268**, 25463–25468.

TANIZAWA A, FUJIMORI A, FUJIMORI Y AND POMMIER Y. (1994). Comparison of topoisomerase I inhibition, DNA damage, and cytotoxicity of camptothecin derivatives presently in clinical trials. *J. Natl Cancer Inst.*, **86**, 836–842.

TEN BOKKEL HUININK WW, RODENHUIS S, BEIJNEN J, DUBBELMAN R AND KOIER I. (1992). Phase I study of the topoisomerase I inhibitor topotecan (SK & F 104864-A). (abstract 260). *Proc. Am. Soc. Clin. Oncol.*, **11**, 110.

TSURUO T, MATSUZAKI T, MATSUSHITA M, SAITO H AND YOKOKURA T. (1988). Antitumor effect of CPT-11, a new derivative of camptothecin, against pleiotropic drug-resistant tumors *in vitro* and *in vivo*. *Cancer Chemother. Pharmacol.*, **21**, 71–74.

TUBERGEN D, PRATT C, STEWART C AND VIETTI T. (1994). Phase I study of topotecan in children with refractory solid tumors: a pediatric oncology group study. (abstract 463). *Proc. Am. Soc. Clin. Oncol.*, **13**, 167.

VAN DER ZEE AG, DEJONG S, KEITH WN, HOLLEMA H, BOONSTRA H AND DE VRIES EG. (1991). P-glycoprotein expression and DNA topoisomerase I and II activity in benign tumors of the ovary and in malignant tumors of the ovary, before and after platinum/cyclophosphamide chemotherapy. *Cancer Res.*, **51**, 5915–5920.

VERWEIJ J, LUND B, BEIJNEN J, PLANTING A, DE BOER-DENNERT M, KOIER I, ROSING H AND HANSEN H. (1993). Phase I and pharmacokinetics study of topotecan, a new topoisomerase I inhibitor. *Ann. Oncol.*, **4**, 673–678.

WAGENER DJTH, WANDERS J, DIRIX LY, CATIMEL G, SIEGENTHALER P, FRANKLIN H AND VERWEIJ J. (1994). Phase II trial of CPT-11 in patients with advanced pancreatic cancer. (suppl.5, abstract 459). *Ann. Oncol.*, **5**, 190.

WALL ME, WANI MC, COOK CE, PALMER KH, MCPHAIL AT AND SIM GA. (1966). A. Plant antitumor agents. I. The isolation and structure of camptothecin, a novel alkaloidal leukemia and tumor inhibitor from *Camptotheca acuminata*. *J. Am. Chem. Soc.*, **88**, 3888–3889.

WALL JG, BURRIS HA, VON HOFF DD, RODRIGUEZ G, KNEUPER-HALL R, SHAFFER D, O'ROURKE T, BROWN T, WEISS G, CLARK G AND ET AL. (1992). A phase I clinical and pharmacokinetic study of the topoisomerase I inhibitor topotecan (SK&F 104864) given as an intravenous bolus every 21 days. *Anti-Cancer Drugs*, **3**, 337–345.

WALL ME, WANI MC, NICHOLAS AW, MANIKUMAR G, TELE C, MOORE L, TRUESDALE A, LEITNER P AND BESTERMAN JM. (1993). Plant antitumor agents. 30. Synthesis and structure activity of novel camptothecin analogues. *J. Med. Chem.*, **36**, 2689–2700.

WALTON MI, WHYSONG D, O'CONNOR PM, HOCKENBERY D, KORSMEYER SJ AND KOHN KW. (1993). Constitutive expression of human Bcl-2 modulates nitrogen mustard and camptothecin induced apoptosis. *Cancer Res.*, **53**, 1853–1861.

WANDERS J, ARDIZZONI A, HANSEN HH, DOMBERNOWSKY P, POSTMUS PE, BUITENHUIS M, MCDONALD M, GIACCONE G, VERWEIJ J. on behalf of the EORTC-ECTG and EORTC-LCCG. (1995). Phase II study of topotecan in refractory and sensitive small cell lung cancer. (abstract 1415). *Proc. Am. Soc. Clin. Oncol.*, **36**, 237.

WANI MC, RONMAN PE, LINDLEY JT AND WALL ME. (1980). Plant antitumor agents. 18. Synthesis and biological activity of camptothecin analogues. *J. Med. Chem.*, **23**, 554–560.

WANI MC, NICHOLAS AW, MANIKUMAR G AND WALL ME. (1987). Plant antitumor agents. 25. Total synthesis and antileukemic activity of ring A substituted camptothecin analogues. Structure–activity correlations. *J. Med. Chem.*, **30**, 1774–1779.

WEITZ JJ, JUNG S-H, MARSCHKE RF, Jr, FITCH TR AND JETT JR. (1995). Randomized phase II trial of two schedules of topotecan for the treatment of advanced stage non-small cell lung carcinoma (NSCLC): a North Central Cancer Treatment Group (NCCTG) trial. (abstract 1053). *Proc. Am. Soc. Clin. Oncol.*, **14**, 348.

WOESSNER RD, ENG WK, HOFMANN GA, RIEMAN DJ, MCCABE FL, HERTZBERG RP, MATTERN MR, TAN KB AND JOHNSON RK. (1992). Camptothecin hyper-resistant P388 cells: drug-dependent reduction in topoisomerase I content. *Oncol. Res.*, **4**, 481–488.

ZHANG H, D'ARPA P AND LIU LF. (1990). A model for tumor cell killing by topoisomerase poisons. *Cancer Cells*, **2**, 23–27.